# Explainable machine learning reveals threshold associations and distinct phenotypes linking migraine features to white matter hyperintensities in women with episodic migraine

**DOI:** 10.1186/s10194-026-02488-8

**Published:** 2026-08-04

**Authors:** Qingling Yang, Sen Deng, Jing Qin, Harry Haoxiang Wang, Kin Cheung, Yao Jie Xie

**Affiliations:** 1 School of Nursing, The Hong Kong Polytechnic University, Hung Hom, Kowloon, Hong Kong, China; 2 School of Public Health, Sun Yat-Sen University, Guangzhou, China; 3 College of Medicine and Veterinary Medicine, The University of Edinburgh, Edinburgh, UK; 4 Research Centre for Chinese Medicine Innovation, The Hong Kong Polytechnic University, Hong Kong, China; 5 Joint Research Centre for Primary Health Care, The Hong Kong Polytechnic University, Hong Kong, China

**Keywords:** Migraine, White matter hyperintensities, Cerebral small vessel disease, Explainable machine learning, SHapley Additive exPlanations, Aura, Phenotyping

## Abstract

**Background:**

White matter hyperintensities (WMHs) are prevalent in migraine, yet their relationship to specific migraine features remains poorly defined. Prior studies have used coarse phenotyping and linear methods, leaving potential threshold effects and the broader migraine phenotype unexplored. We applied an explainable machine-learning framework to characterise associations between migraine features and WMH volume and to identify data-driven phenotypes.

**Methods:**

Baseline data from two ongoing randomised controlled trials in Hong Kong Chinese women with episodic migraine were used for analyses. Participants underwent 3T brain magnetic resonance imaging (MRI) and WMH were quantified from manually corrected segmentations by radiologists. Migraine features (attack frequency, duration, pain intensity, aura subtypes, accompanying symptoms, triggers, and acute medication use) were derived from prospective three-month diaries and structured interviews. An XGBoost model was developed with nested five-fold cross-validation and interpreted using SHapley Additive exPlanations (SHAP). Unsupervised K-means clustering on patient-level SHAP values identified phenotypes.

**Results:**

The analytic sample comprised 171 women (median age 52.0 years [IQR 38.5–60.0]) with a median WMH volume of 0.90 mL (IQR 0.56–1.31). The model explained modest variance (R² = 0.221) and achieved an area under the curve of 0.801 (95% CI 0.726–0.876) for classifying high WMH burden. SHAP dependence analyses revealed several non-linear patterns of migraine features on WMH. Attack frequency and duration showed apparent saturation, plateauing above approximately 5 attacks/month and 24 h/month, respectively; pain intensity showed an L-shaped threshold pattern above a Numerical Rating Scale score of approximately 5. Somatosensory aura was associated with higher WMH volume. Three phenotypes emerged: Age-Dominant (*n* = 16; older, low burden, low WMH), Cumulative Risk (*n* = 59; older with high vascular and migraine burden, highest WMH volume), and Migraine-Dominant (*n* = 96; younger, migraine as the primary driver, intermediate WMH).

**Conclusions:**

Explainable machine learning revealed threshold associations, including saturation patterns for attack frequency and duration and an L-shaped pattern for pain intensity, together with a pattern linking somatosensory aura to higher WMH volume, which may help reconcile longstanding discrepancies in the migraine-WMH literature. Findings from this study are hypothesis-generating and warrant validation in larger, longitudinal, and multi-ethnic cohorts.

**Supplementary Information:**

The online version contains supplementary material available at https://doi.org/10.1186/s10194-026-02488-8.

## Introduction

Migraine is one of the most prevalent neurological disorders, affecting more than one billion individuals worldwide and ranking among the leading causes of years lived with disability, particularly in women [1, 2]. Beyond the burden of recurrent attacks, neuroimaging studies have consistently demonstrated that migraine is associated with a higher prevalence of cerebral white matter hyperintensities (WMHs) [3–5]. WMHs are signal abnormalities on brain magnetic resonance imaging (MRI) that reflect a spectrum of underlying pathology, including demyelination, gliosis, and small-vessel ischemic changes [6]. In the general population, greater WMH burden has been linked to an increased risk of stroke, accelerated cognitive decline, and dementia [7]. Clarifying the factors that contribute to WMH accumulation in patients with migraine is therefore of considerable clinical importance.

Several studies have examined whether specific migraine features are associated with WMH burden, with most attention focused on attack frequency and aura status; however, findings have been inconsistent [3, 4, 8–11]. A positive, frequency-dependent association between attack frequency and deep WMH load has been reported in some population-based and clinic-based studies [3, 8], whereas others have found no such association after accounting for vascular risk factors [9, 10]. Similarly, an early meta-analysis suggested that migraine with aura conferred a greater WMH risk [4], but subsequent studies with more rigorous covariate adjustment have not consistently replicated this finding [9, 11]. A likely explanation for these discrepancies is the reliance on coarse clinical phenotyping (such as dichotomous aura classification or broad frequency categories), which may mask complex non-linear relationships [3, 4, 8]. Moreover, a wide range of potentially relevant migraine features, including specific accompanying symptoms (e.g., photophobia, nausea, and other sensory or autonomic manifestations), individual trigger profiles, and aura subtypes, has rarely been examined in relation to WMH, leaving a substantial portion of the migraine phenotype unexplored.

From an analytical standpoint, prior research in this area has relied predominantly on conventional regression models [3, 4, 8–11]. While appropriate for estimating population-level average associations, these methods presuppose linear and additive relationships among predictors, assumptions that may be overly restrictive when non-linear relationships and complex interactions are biologically plausible [12]. Moreover, conventional regression approaches yield only group-level estimates and cannot quantify the contribution of each predictor at the level of an individual patient, an increasingly relevant objective in the context of precision medicine [13].

Explainable machine learning offers an approach well suited to addressing these limitations. Tree-based ensemble algorithms such as eXtreme Gradient Boosting (XGBoost) [14] can accommodate non-linear effects and complex interactions among predictors without a priori parametric assumptions, while SHapley Additive exPlanations (SHAP) [15], a model-agnostic interpretability method grounded in cooperative game theory, provides both global importance rankings and individualised attribution profiles that quantify the direction and magnitude of each variable’s contribution for every observation. This combination enables not only the identification of population-level associations, but also the detection of patient-level heterogeneity that conventional approaches may overlook.

In this study, we integrated demographic, clinical variables, and comprehensive migraine features derived from prospective three-month diary records and structured clinical interviews, with quantitative MRI-based WMH volumetry, in a dataset of women with episodic migraine in Hong Kong. Using an explainable machine-learning approach combining XGBoost with SHAP, we aimed to: (1) quantify the incremental contribution of migraine-specific features to WMH volume beyond established vascular risk factors; (2) characterise the direction, magnitude, and shape (including non-linear patterns) of these associations at the population and individual level; and (3) identify data-driven patient phenotypes defined by the dominant drivers of WMH burden.

## Methods

### Study sources and participants

Data were extracted from baseline assessments of two ongoing randomised controlled trials (RCTs) focusing on women with episodic migraine (ClinicalTrials.gov Identifiers: NCT05690737, registered on 19 January 2023; NCT06967909, registered on 13 May 2025; protocol published previously [16]). Eligible participants were female residents aged 18 years or older with a clinical diagnosis of migraine, with or without aura, in accordance with the International Classification of Headache Disorders, 3rd edition (ICHD-3) [17]. Participants were excluded if they presented with secondary headaches or other neurological disorders affecting brain morphology (e.g., brain tumors or epilepsy), including space-occupying lesions identified on study MRI, or if their non-migraine headache frequency exceeded five days per month.

At the time of the present analysis (September 2025), 173 eligible participants had completed baseline clinical assessments and brain MRI prior to intervention. Two participants were excluded owing to incidental meningiomas identified on brain MRI, yielding a final analytic sample of 171 women. Recruitment for the two parent trials commenced in September 2023 and was ongoing at the time of analysis. No a priori sample-size calculation was performed for this secondary analysis; the study included all eligible participants who had completed baseline brain MRI and clinical assessment by September 2025. The study was approved by the Institutional Review Board of The Hong Kong Polytechnic University (Reference: HSEARS20220721006; HSEARS20240312017), and all participants provided written informed consent. The study was reported in accordance with the STrengthening the Reporting of OBservational studies in Epidemiology (STROBE) guidelines for cross-sectional studies [18].

### Migraine feature assessment

Migraine features were derived from a prospective migraine diary maintained for three consecutive months, with the recording period spanning the date of the baseline MRI scan and completed before commencement of the trial intervention. From this diary, we computed mean monthly values for attack frequency (attacks/month) and mean pain intensity (0–10 Numerical Rating Scale [NRS]). Attack duration was recorded per attack (hours) and aggregated to the mean cumulative monthly duration (hours/month), consistent with the monthly scale used for attack frequency. Acute medication use was likewise quantified from the diary. Aura subtypes (e.g., visual, somatosensory, motor, and cognitive), accompanying symptoms (e.g., photophobia, phonophobia, nausea, vomiting, and neck stiffness or fatigue), and trigger factors (e.g., dietary, stress, physical activity, and hormonal) were ascertained by integrating the prospective three-month diary with a structured clinical interview addressing migraine experience over the preceding month, with a feature coded as present if endorsed by either source. The interview was administered by a trained research assistant, and both sources used a symptom checklist in which specific individual symptoms were probed within each aura subtype rather than recording subtype status alone.

### Brain MRI acquisition and WMH quantification

Brain MRI was performed on a 3.0 T Siemens MAGNETOM Prisma scanner (32-channel head coil) at the University Research Facility in Behavioral and Systems Neuroscience, The Hong Kong Polytechnic University. The protocol comprised a three-dimensional T1-weighted MPRAGE sequence (voxel size 0.8 × 0.8 × 0.8 mm³; TR/TE/TI = 2500/2.22/1120 ms; flip angle 8°) and a three-dimensional T2-FLAIR SPACE sequence with compressed sensing (voxel size 0.9 × 0.9 × 0.9 mm³; TR/TE/TI = 7000/395/2050 ms).

WMH were segmented on T2-FLAIR images using a human-in-the-loop deep learning approach based on the nnU-Net framework [19]. First, two radiologists independently performed manual WMH segmentation on all 30 MRI scans, and a third senior neuroradiologist adjudicated discrepancies to establish a consensus reference standard. These 30 manually segmented cases were used to train and compare four segmentation methods under nested five-fold cross-validation, and the best-performing model (nnU-Net) was selected as the assistive tool (Supplementary Fig. 1). This model then generated initial predictions for the remaining 141 scans, each of which was subsequently reviewed and manually corrected by a separate senior neuroradiologist. Agreement between the model-generated masks and the corresponding radiologist-corrected reference for these 141 scans was high (mean Dice = 0.945, SD 0.068). Consequently, the WMH masks used for all analyses were either fully manual (*n* = 30) or model-assisted but manually corrected (*n* = 141); no uncorrected automated output was used. Total WMH volume was calculated as the sum of segmented voxels multiplied by voxel volume. Total intracranial volume (TIV) was estimated from T1-weighted images via automated segmentation. WMH volume (mL) was log-transformed prior to analysis owing to its right-skewed distribution (Supplementary Fig. 2). TIV (mL) was also log-transformed for consistency in the scale of volumetric measures.

### Demographic and clinical covariates

Demographic, anthropometric, lifestyle, reproductive, and medical history data were collected through standardised questionnaires at baseline. In addition, validated instruments were administered to assess migraine-related disability (Migraine Disability Assessment [MIDAS] [20]; Headache Impact Test-6 [HIT-6] [21]), quality of life (Migraine-Specific Quality of Life Questionnaire [MSQ] [22]), sleep quality (Pittsburgh Sleep Quality Index [PSQI] [23]), perceived stress (Perceived Stress Scale [PSS] [24]), fatigue, and physical activity. These variables, together with the migraine features described above, constituted the candidate predictors considered in subsequent feature selection and modelling (Table 1).

**Table 1:** Characteristics of participants stratified by white matter hyperintensity volume

	Overall	WMH volume		*P* value
		< median	≥median	
**No. of Participants**	171	85	86	
**Demographics**				
Age (years)	52.00 (38.50, 60.00)	47.00 (32.00, 57.00)	57.00 (46.50, 62.00)	< 0.001
Number of children	0.00 (0.00, 2.00)	0.00 (0.00, 2.00)	1.00 (0.00, 2.00)	0.25
Household size	2.00 (1.00, 3.00)	2.00 (1.00, 3.00)	2.00 (1.00, 2.75)	0.77
Married, n (%)	98 (57.3)	41 (48.2)	57 (66.3)	0.03
Tertiary education, n (%)	106 (62.0)	56 (65.9)	50 (58.1)	0.38
Employed, n (%)	83 (48.5)	38 (44.7)	45 (52.3)	0.40
Monthly household income, (1000 HKD)	30.00 (20.00, 36.57)	31.67 (25.00, 40.00)	26.47 (20.00, 35.00)	0.005
**Anthropometrics**				
Body mass index (kg/m²)	22.10 (20.20, 25.26)	21.73 (20.19, 24.34)	22.80 (20.36, 25.41)	0.24
Waist circumference (cm)	76.50 (71.35, 85.10)	75.00 (71.00, 83.00)	79.05 (73.00, 86.50)	0.05
Hip circumference (cm)	94.50 (89.75, 100.25)	95.00 (89.50, 100.50)	94.00 (90.00, 100.00)	0.94
Thigh circumference (cm)	53.00 (49.00, 56.00)	53.10 (50.00, 56.00)	52.60 (48.55, 55.88)	0.50
Percent body fat (%)	33.10 (28.70, 37.00)	32.80 (27.40, 37.10)	33.20 (28.83, 36.72)	0.58
Systolic blood pressure (mmHg)	115.00 (107.00, 126.00)	112.00 (104.50, 122.00)	121.00 (108.12, 131.75)	0.002
Diastolic blood pressure (mmHg)	72.50 (65.50, 79.75)	71.00 (65.00, 75.00)	74.00 (68.00, 83.38)	0.001
**Lifestyle Factors**				
Alcohol consumption, n (%)	11 (6.4)	4 (4.7)	7 (8.1)	0.55
Current smoker, n (%)	4 (2.3)	1 (1.2)	3 (3.5)	0.62
Coffee consumption, n (%)	109 (63.7)	51 (60.0)	58 (67.4)	0.39
Tea consumption, n (%)	137 (80.1)	65 (76.5)	72 (83.7)	0.32
Vigorous activity (MET-min/week)	0.00 (0.00, 480.00)	0.00 (0.00, 0.00)	0.00 (0.00, 480.00)	0.20
Moderate activity (MET-min/week)	0.00 (0.00, 360.00)	0.00 (0.00, 240.00)	40.00 (0.00, 480.00)	0.14
Walking (MET-min/week)	1039.50 (693.00, 2079.00)	990.00 (693.00, 1386.00)	1097.25 (693.00, 2772.00)	0.37
Total physical activity (MET-min/week)	1653.00 (919.50, 3292.50)	1386.00 (754.00, 2439.00)	1963.50 (990.00, 3711.75)	0.12
Sedentary time (min/day)	540.00 (360.00, 720.00)	600.00 (360.00, 720.00)	525.00 (300.00, 660.00)	0.16
Daily fruit intake (servings)	1.00 (0.50, 1.50)	1.00 (0.50, 1.50)	1.00 (0.50, 1.38)	0.93
Daily vegetable intake (servings)	1.00 (0.50, 2.00)	1.00 (0.50, 2.00)	1.00 (0.50, 1.88)	0.73
Daily whole grain intake (servings)	1.00 (1.00, 2.00)	1.10 (1.00, 2.00)	1.00 (1.00, 2.00)	0.54
**Reproductive History**				
Age at menarche (years)	12.00 (11.00, 14.00)	12.00 (11.00, 13.00)	13.00 (12.00, 14.00)	0.11
History of infertility, n (%)	28 (16.4)	12 (14.1)	16 (18.6)	0.56
Attempted pregnancy, n (%)	87 (50.9)	49 (57.6)	38 (44.2)	0.11
Contraceptive use, n (%)	139 (81.3)	70 (82.4)	69 (80.2)	0.87
Hormonal medication use, n (%)	4 (2.3)	2 (2.4)	2 (2.3)	> 0.99
Diagnosed infertility, n (%)	8 (4.7)	4 (4.7)	4 (4.7)	> 0.99
History of preterm birth, n (%)	27 (15.8)	9 (10.6)	18 (20.9)	0.10
History of amenorrhea, n (%)	32 (18.7)	13 (15.3)	19 (22.1)	0.35
History of miscarriage, n (%)	16 (9.4)	8 (9.4)	8 (9.3)	> 0.99
History of stillbirth, n (%)	17 (9.9)	8 (9.4)	9 (10.5)	> 0.99
History of abortion, n (%)	2 (1.2)	1 (1.2)	1 (1.2)	> 0.99
Postmenopausal status, n (%)	85 (49.7)	31 (36.5)	54 (62.8)	0.001
Hormone replacement therapy, n (%)				0.18
Never	156 (91.2)	80 (94.1)	76 (88.4)	
Past	12 (7.0)	3 (3.5)	9 (10.5)	
Current	3 (1.8)	2 (2.4)	1 (1.2)	
**Medical History**				
Hypertension, n (%)	31 (18.1)	7 (8.2)	24 (27.9)	0.002
Diabetes mellitus, n (%)	14 (8.2)	6 (7.1)	8 (9.3)	0.80
Hyperlipidemia, n (%)	44 (25.7)	18 (21.2)	26 (30.2)	0.24
Cardiovascular disease, n (%)	7 (4.1)	3 (3.5)	4 (4.7)	> 0.99
Angina pectoris, n (%)	2 (1.2)	1 (1.2)	1 (1.2)	> 0.99
Stroke, n (%)	5 (2.9)	3 (3.5)	2 (2.3)	0.99
Deep vein thrombosis, n (%)	13 (7.6)	3 (3.5)	10 (11.6)	0.09
Chronic lung disease, n (%)	0 (0)	0 (0)	0 (0)	-
Liver disease, n (%)	1 (0.6)	1 (1.2)	0 (0.0)	> 0.99
Breast cancer, n (%)	8 (4.7)	2 (2.4)	6 (7.0)	0.29
Ovarian cancer, n (%)	0 (0)	0 (0)	0 (0)	-
Uterine cancer, n (%)	1 (0.6)	0 (0.0)	1 (1.2)	> 0.99
Family history of migraine, n (%)	110 (64.3)	51 (60.0)	59 (68.6)	0.31
Family history of hypertension, n (%)	116 (67.8)	55 (64.7)	61 (70.9)	0.48
Family history of diabetes, n (%)	68 (39.8)	34 (40.0)	34 (39.5)	> 0.99
Family history of hyperlipidemia, n (%)	71 (41.5)	36 (42.4)	35 (40.7)	0.95
Family history of CVD, n (%)	27 (15.8)	14 (16.5)	13 (15.1)	0.97
Family history of stroke, n (%)	31 (18.1)	13 (15.3)	18 (20.9)	0.45
Family history of lung disease, n (%)	23 (13.5)	9 (10.6)	14 (16.3)	0.39
Family history of liver disease, n (%)	28 (16.4)	15 (17.6)	13 (15.1)	0.81
Family history of breast cancer, n (%)	8 (4.7)	2 (2.4)	6 (7.0)	0.29
Family history of ovarian cancer, n (%)	11 (6.4)	5 (5.9)	6 (7.0)	> 0.99
Family history of uterine cancer, n (%)	1 (0.6)	0 (0.0)	1 (1.2)	> 0.99
Family history of colon cancer, n (%)	3 (1.8)	1 (1.2)	2 (2.3)	> 0.99
**Migraine Features**				
Frequency (attacks/month)	3.00 (1.67, 5.00)	2.33 (1.33, 5.00)	3.17 (2.33, 5.00)	0.01
Intensity (0–10)	4.29 (3.33, 5.80)	4.17 (3.00, 5.40)	4.48 (3.51, 6.33)	0.08
Duration (hours)	5.92 (3.00, 13.63)	5.78 (3.00, 14.33)	5.96 (3.05, 12.84)	0.96
*** Aura symptoms***				
Visual aura, n (%)	84 (49.1)	40 (47.1)	44 (51.2)	0.70
Somatosensory aura, n (%)	73 (42.7)	28 (32.9)	45 (52.3)	0.02
Motor aura, n (%)	29 (17.0)	13 (15.3)	16 (18.6)	0.71
Cognitive aura, n (%)	48 (28.1)	22 (25.9)	26 (30.2)	0.64
*** Accompanying symptoms***				
Photophobia, n (%)	118 (69.0)	59 (69.4)	59 (68.6)	> 0.99
Phonophobia, n (%)	102 (59.6)	49 (57.6)	53 (61.6)	0.71
Nausea, n (%)	132 (77.2)	70 (82.4)	62 (72.1)	0.16
Vomiting, n (%)	80 (46.8)	39 (45.9)	41 (47.7)	0.94
Numbness, n (%)	34 (19.9)	12 (14.1)	22 (25.6)	0.09
Muscle weakness, n (%)	54 (31.6)	21 (24.7)	33 (38.4)	0.08
Hyperactivity, n (%)	4 (2.3)	2 (2.4)	2 (2.3)	> 0.99
Hypoactivity, n (%)	95 (55.6)	51 (60.0)	44 (51.2)	0.313
Speech difficulty, n (%)	14 (8.2)	4 (4.7)	10 (11.6)	0.17
Repetitive yawning, n (%)	85 (49.7)	45 (52.9)	40 (46.5)	0.49
Visual disturbance, n (%)	75 (43.9)	40 (47.1)	35 (40.7)	0.49
Food cravings, n (%)	19 (11.1)	10 (11.8)	9 (10.5)	0.98
Depressed mood, n (%)	113 (66.1)	59 (69.4)	54 (62.8)	0.45
Neck stiffness/fatigue, n (%)	147 (86.0)	67 (78.8)	80 (93.0)	0.01
*** Trigger factors***				
Dietary triggers	96 (56.1)	50 (58.8)	46 (53.5)	0.58
Sleep triggers	163 (95.3)	82 (96.5)	81 (94.2)	0.73
Stress triggers	144 (84.2)	76 (89.4)	68 (79.1)	0.10
Physical activity triggers	73 (42.7)	35 (41.2)	38 (44.2)	0.81
Environmental triggers	163 (95.3)	80 (94.1)	83 (96.5)	0.71
Hormonal triggers	105 (61.4)	57 (67.1)	48 (55.8)	0.18
Acute medication use	114 (66.7)	55 (64.7)	59 (68.6)	0.71
**Clinical Scores**				
MSQ score	72.62 (63.10, 80.95)	71.43 (64.29, 78.57)	72.62 (63.10, 81.85)	0.74
MIDAS score	9.00 (4.00, 19.25)	9.00 (4.00, 21.00)	8.75 (4.00, 17.88)	0.75
HIT-6 score	59.00 (53.00, 63.00)	59.00 (54.00, 63.00)	58.00 (52.25, 62.00)	0.15
PSQI score	16.00 (15.00, 18.00)	16.00 (15.00, 18.00)	16.00 (15.00, 18.00)	0.74
Fatigue score	6.00 (5.00, 7.00)	6.00 (5.00, 7.00)	6.00 (5.00, 7.00)	0.74
Perceived stress score	37.00 (29.00, 43.00)	37.00 (30.00, 41.00)	36.50 (29.00, 43.00)	0.80
**Neuroimaging**				
WMH volume (mL)	0.90 (0.56, 1.31)	0.56 (0.39, 0.71)	1.31 (1.09, 2.18)	< 0.001
Total intracranial volume (mL)	1380 (1313, 1451)	1366 (1300, 1437)	1408 (1326, 1466)	0.07

Data are presented as median (interquartile range) for continuous variables and n (%) for categorical variables. P values were calculated using the Wilcoxon rank sum test for continuous variables and the chi-square test or Fisher’s exact test for categorical variables. These comparisons are descriptive and were not adjusted for multiple testing; the reported P values should therefore be interpreted as exploratory rather than confirmatory

Abbreviations: WMH, white matter hyperintensity; BMI, body mass index; MSQ, Migraine-Specific Quality of Life Questionnaire; MIDAS, Migraine Disability Assessment; HIT-6, Headache Impact Test-6; PSQI, Pittsburgh Sleep Quality Index; PSS, Perceived Stress Scale

### Statistical analysis

#### Data preprocessing

Missing values were imputed using random forest-based imputation (missForest) (Supplementary Table 1) [25]. Categorical variables with near-zero variance (frequency ratio > 19) were excluded prior to modelling (Supplementary Table 2).

#### Feature selection

Feature selection proceeded in two stages. First, the Boruta algorithm [26] was applied to all non-migraine candidate variables, and those classified as “confirmed” or “tentative” were retained. Migraine-specific features were retained a priori to ensure comprehensive evaluation of the migraine phenotype, as was log-transformed total intracranial volume (TIV) to account for inter-individual variation in head size; both were exempted from this step. Second, pairwise Spearman correlations were computed among the retained variables; where |r| ≥ 0.80, the variable with the weaker univariable Spearman correlation with WMH volume was removed. Variance inflation factors (all < 5) confirmed the absence of problematic multicollinearity.

#### Model development and evaluation

Four models were developed and compared using nested five-fold cross-validation (grid-search hyperparameter tuning in the inner loop; performance estimation in the outer loop). A Full XGBoost model [14] incorporating all candidate predictors was fitted first. Features were then ranked by mean absolute SHAP value [27], and those cumulatively accounting for approximately 85% of total SHAP importance were retained to train a Selected XGBoost model. To test whether a simpler parametric model performed comparably, a Selected Linear Regression model with the same predictors was fitted. Finally, to quantify the incremental contribution of migraine-specific features, a Basic XGBoost model including only the non-migraine predictors from the selected set was fitted. Performance was assessed by mean absolute error (MAE), root mean square error (RMSE), and R², averaged across the five outer folds. Predictive performance was further examined by comparing predicted versus observed log-transformed WMH volume across quartiles (test for linear trend and Pearson correlation) and by the area under the receiver operating characteristic curve (AUC; 95% CI) for classifying high WMH burden (≥ 75th percentile).

#### Model interpretation

Global feature importance was assessed by mean absolute SHAP value. Throughout, SHAP values were interpreted as predictive associations within the fitted model rather than as causal effects or direct indices of biological mechanism. SHAP dependence plots characterised the functional form of each predictor’s marginal association with log-transformed WMH volume, with generalised additive model (GAM) smoothing curves and 95% confidence intervals overlaid for continuous variables. Pairwise SHAP interaction values were computed to quantify effect modification [28].

#### Sensitivity analyses

Four analyses evaluated the robustness of the associations identified by the SHAP-based framework. First, a multivariable linear regression model including all selected predictors was fitted, with restricted cubic splines (RCS) applied to each continuous predictor [29]. For each predictor, an overall likelihood-ratio test assessed its total association with log-transformed WMH volume, and a non-linearity test compared the full spline model with a nested linear-only model; adjusted spline curves were plotted for predictors with significant non-linearity. Univariable linear regression was also performed for each predictor. Second, piecewise linear regression was applied to continuous migraine-specific features showing apparent non-linearity, with candidate breakpoints informed by the SHAP dependence and GAM curves. Each feature was divided into an active zone (steep slope) and a plateau zone (flattened slope); zone-specific slopes were estimated separately, slope reduction was calculated as 1 − |plateau slope / active slope|, and fit was compared with a single linear model over the full range using the Akaike information criterion (AIC). Third, because feature selection was performed once on the full sample, we assessed whether this had introduced data leakage into the final model. The entire modelling workflow, comprising missForest imputation, near-zero-variance filtering, the two-stage feature selection, and SHAP-based ranking, was repeated within the training partition of each outer cross-validation fold, with each test fold excluded from all preprocessing, selection, and training steps. Model performance was re-estimated from the aggregated out-of-fold predictions, and the number of folds in which each feature was retained was recorded as a measure of selection stability. Fourth, because baseline data were pooled from two trials, we assessed whether trial source acted as a confounder or effect modifier. Trial source was added as a covariate to the multivariable regression model, and effect modification was examined by adding interaction terms between trial source and each of the key migraine predictors.

#### SHAP-based patient stratification

K-means clustering [30] was performed on the patient-level SHAP value matrix to identify subgroups with distinct prediction-driving profiles. The optimal number of clusters was determined by the elbow method and mean silhouette coefficient, and clusters were visualised via principal component analysis. Between-cluster differences were assessed using the Kruskal-Wallis test or chi-square/Fisher’s exact test. SHAP waterfall plots were generated for one representative participant per cluster, selected to exemplify that cluster’s characteristic contribution profile. The robustness of the clustering was evaluated by the average silhouette width and by cluster-wise bootstrap stability (Jaccard coefficient over 1000 resamples). To assess whether the phenotypes reflected more than an age gradient, the SHAP profiles of participants within a restricted middle-age band were re-clustered and compared with the full-sample assignment. To assess whether they provided information beyond age and migraine severity, participants were also clustered on the raw values of age, attack frequency, attack duration, and mean pain intensity (standardised, k = 3). Agreement between partitions was quantified using the adjusted Rand index.

#### Software

All analyses were conducted in R (version 4.3.3). Two-sided *P* < 0.05 was considered statistically significant.

## Results

### Participant characteristics

The analytic sample comprised 171 women with a median age of 52.0 years (IQR 38.5–60.0) and a median WMH volume of 0.90 mL (IQR 0.56–1.31). Participants with above-median WMH volume were significantly older (*P* < 0.001), more likely to be married (*P* = 0.03), and had lower household income (*P* = 0.005), higher systolic blood pressure (*P* = 0.002) and diastolic blood pressure (*P* = 0.001), higher migraine frequency (*P* = 0.01) and a greater prevalence of somatosensory aura (*P* = 0.02) and neck stiffness or fatigue (*P* = 0.01) (Table 1).

#### Feature selection

Boruta identified age, household income, systolic blood pressure, diastolic blood pressure, deep vein thrombosis history, and menopausal status as confirmed or tentative (Supplementary Fig. 3). Menopausal status was subsequently removed owing to collinearity with age (|r| = 0.82) (Supplementary Fig. 4). All retained variables had VIF < 5 (Supplementary Fig. 5). Together with the a priori-retained migraine-specific features and log-transformed TIV, these variables formed the candidate set for modelling.

### Model performance

An initial full XGBoost model was trained on all candidate predictors, and global feature importance was quantified by mean absolute SHAP values (Supplementary Fig. 6). The top 10 features - age, household income, attack frequency, diastolic blood pressure, total intracranial volume, attack duration, nausea, systolic blood pressure, somatosensory aura, and mean pain intensity - collectively accounted for 84.4% of total SHAP importance. These 10 features were retained for the final model.

**Fig. 1:**
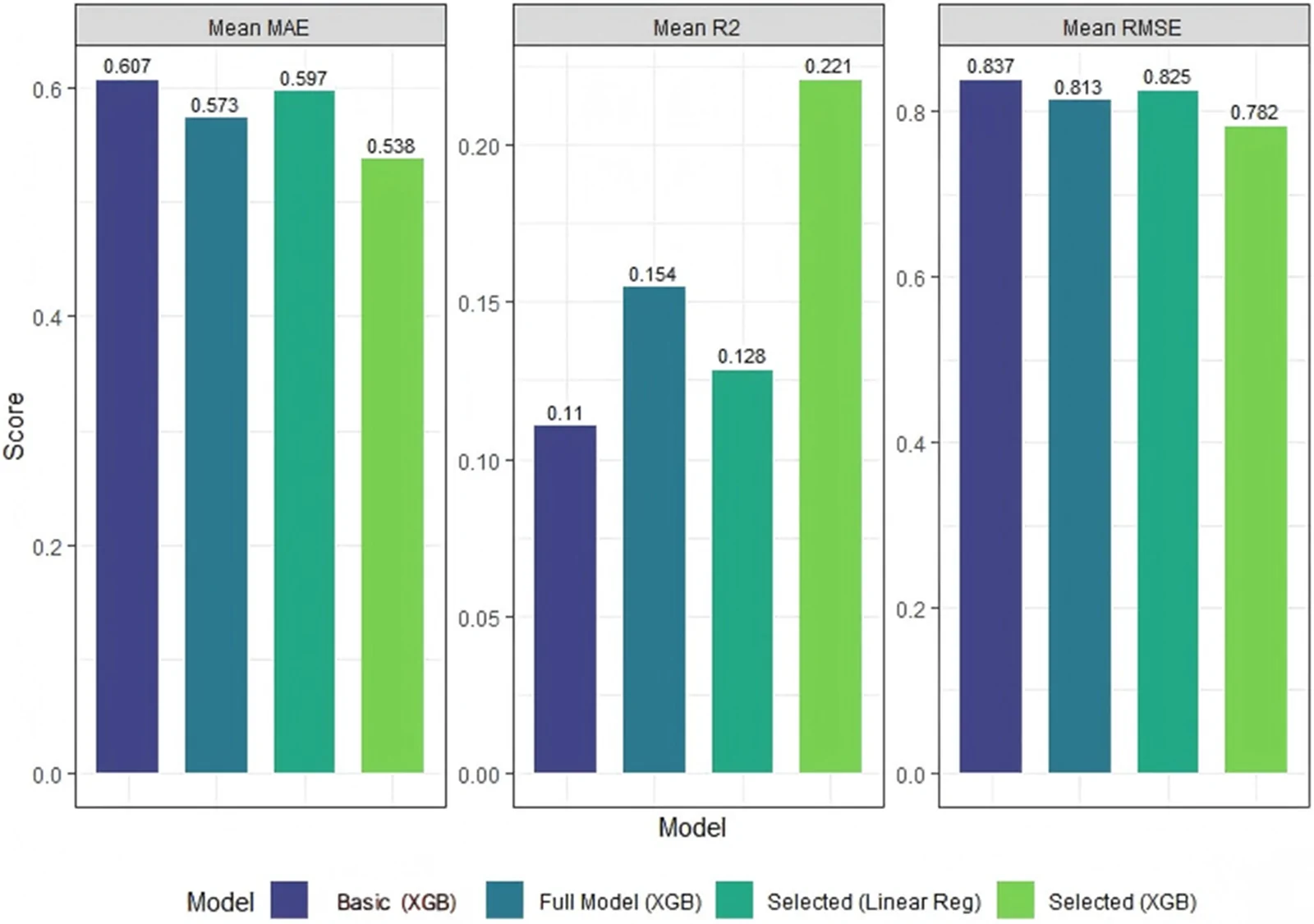
Performance comparison of predictive models for white matter hyperintensity volume. Bars compare the predictive performance of four models evaluated via nested five-fold cross-validation: Basic XGBoost (basic demographic/clinical variables only), Full XGBoost (all candidate variables), Selected Linear Regression (top 10 features), and Selected XGBoost (top 10 features). Performance is measured by Mean Absolute Error (MAE), Root Mean Square Error (RMSE), and the Coefficient of Determination (R²). The Selected XGBoost model (light green) demonstrated superior performance, achieving the lowest error rates and the highest explained variance

The Selected XGBoost model using these 10 features yielded the highest cross-validated R² (R² = 0.221), compared with the Full XGBoost (R² = 0.154), Selected Linear Regression (R² = 0.128), and Basic XGBoost (R² = 0.110) (Fig. 1). The Selected XGBoost was therefore designated as the final model. Predicted values increased monotonically across quartiles of observed log-transformed WMH volume (test for linear trend: *P* < 0.001; Pearson *r* = 0.473; Supplementary Fig. 7), and the model discriminated high WMH burden (≥ 75th percentile) with an AUC of 0.801 (95% CI 0.726–0.876) (Supplementary Fig. 8).

### Feature importance and associations

**Fig. 2:**
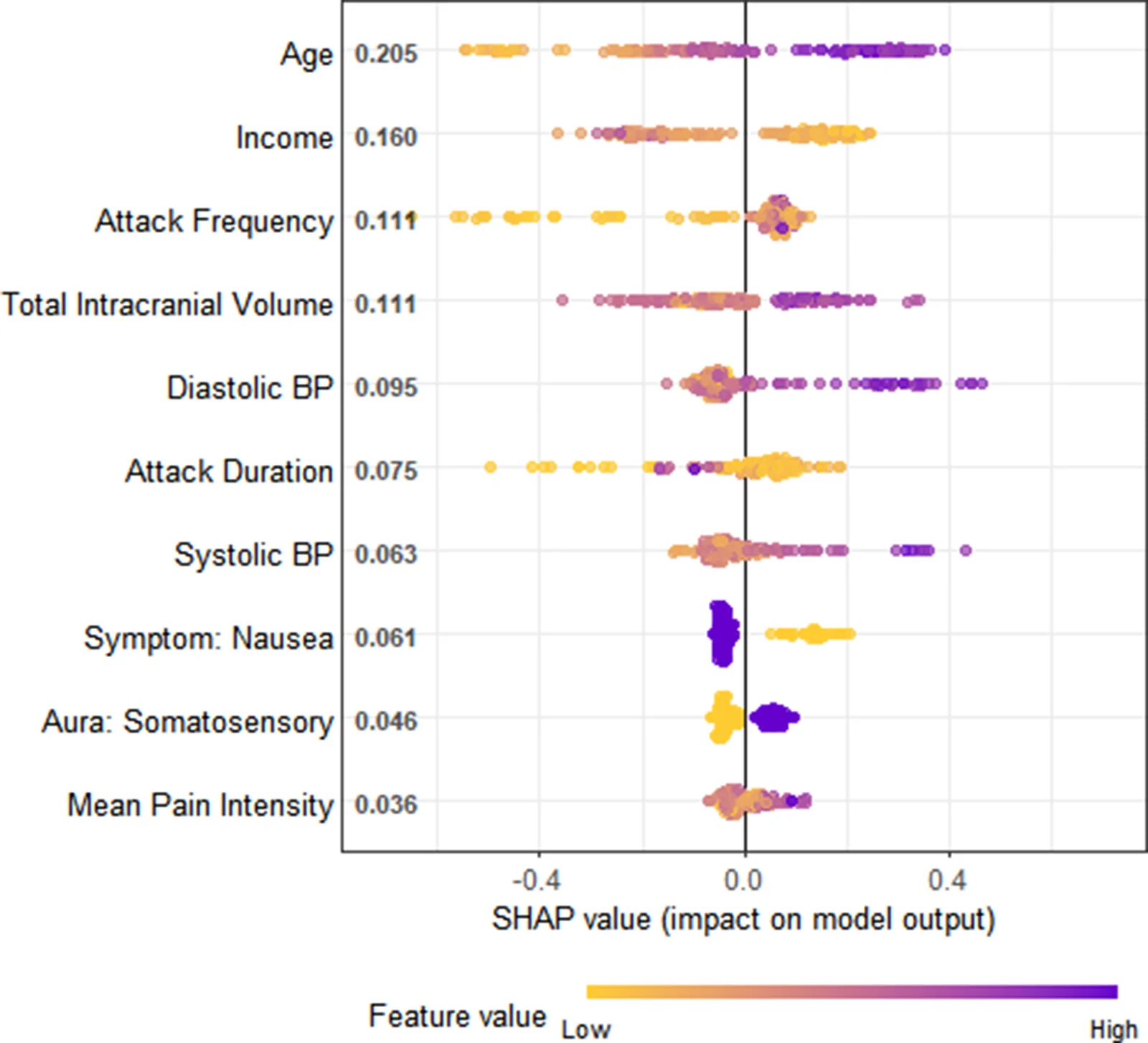
Global feature importance in the final selected XGBoost model. SHapley Additive exPlanations (SHAP) summary plot ranking the predictors of the final selected XGBoost model according to their mean absolute SHAP values. Each dot represents a single patient. The colour gradient indicates the raw feature value, ranging from low (yellow) to high (purple). The x-axis displays the SHAP value, which quantifies the impact of each feature on the model's output; positive values indicate an increased predicted WMH volume. Vertical dispersion (jitter) at a single SHAP value indicates a high density of overlapping data points

In the final model, the global SHAP-based feature importance ranking was broadly consistent with the initial full model, with minor re-ordering among mid-ranked features (Fig. 2). Age remained the most important predictor (mean |SHAP| = 0.205), followed by household income (0.160), attack frequency (0.111), total intracranial volume (0.111), diastolic blood pressure (0.095), attack duration (0.075), systolic blood pressure (0.063), nausea (0.061), somatosensory aura (0.046), and mean pain intensity (0.036).

**Fig. 3:**
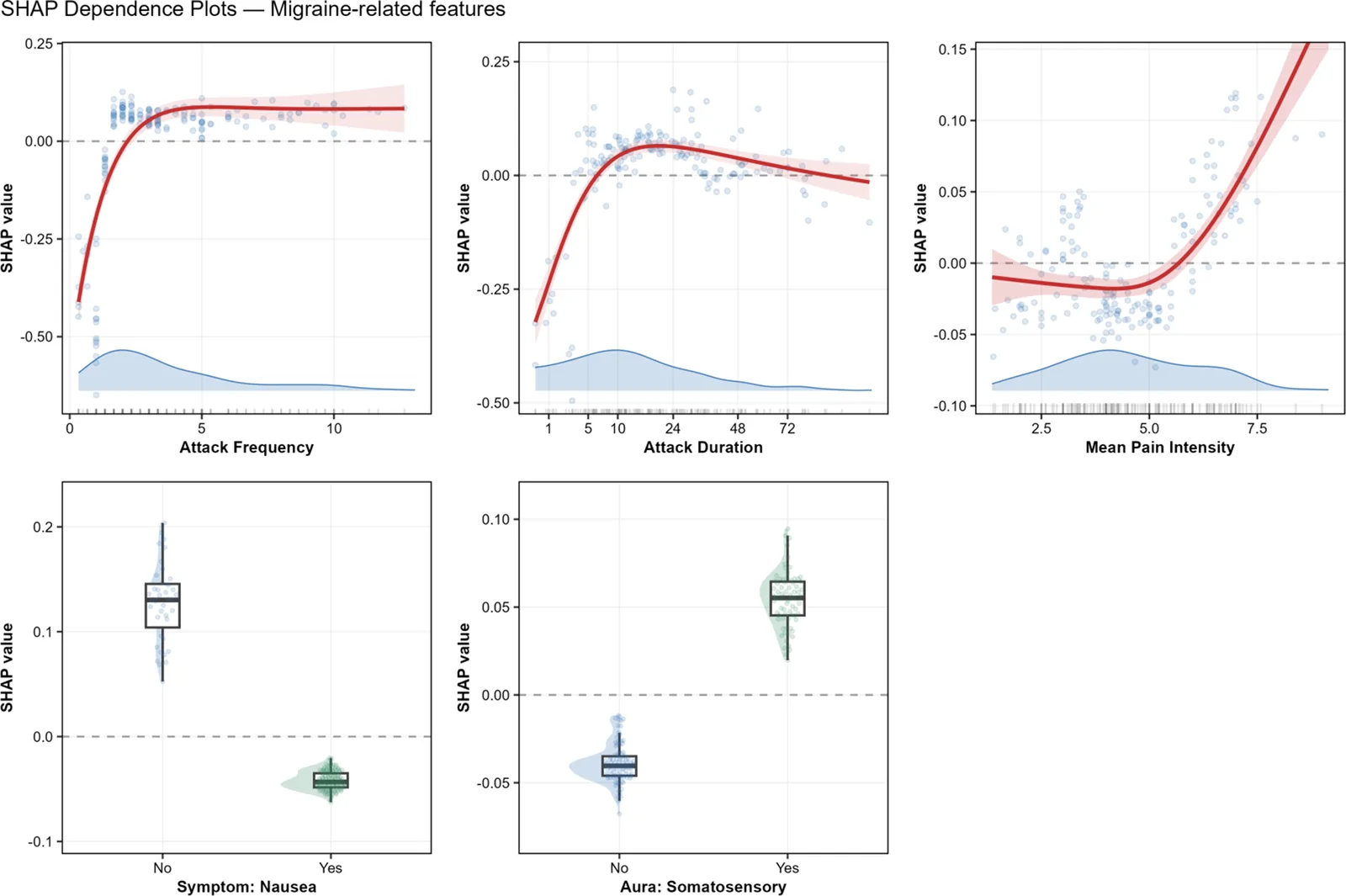
SHAP dependence plots for migraine-specific features and predicted WMH volume. SHAP dependence plots demonstrating the independent predictive contributions of migraine-related features to WMH volume. The y-axis represents the SHAP value (the feature's adjusted contribution to the model output), where values > 0 indicate an increased predicted WMH volume. Solid red lines represent smoothing curves fitted by generalised additive models (GAMs) with 95% confidence intervals. For continuous variables, solid red lines represent fitted smoothing curves with 95% confidence intervals, alongside marginal density distributions (blue shaded areas) and rug marks. For Attack Duration, the x-axis is displayed on a log scale. Box plots illustrate SHAP value distributions for categorical variables.Abbreviations: SHAP, SHapley Additive exPlanations; WMH, white matter hyperintensity

SHAP dependence plots revealed non-linear or threshold-type patterns for several predictors (Supplementary Fig. 9; Fig. 3). Among clinical and demographic variables, age and TIV showed broadly linear positive associations; household income exhibited a steeply declining curve; and both systolic and diastolic blood pressure displayed J-shaped relationships with nadirs in the normotensive range (Supplementary Fig. 9).

Among migraine-specific features, attack frequency and attack duration both demonstrated saturation patterns (Fig. 3). Attack frequency showed a steep positive increase below approximately 5 attacks/month, beyond which the SHAP contribution plateaued. Attack duration exhibited a similar rapid rise below approximately 24 h/month, plateauing thereafter. Mean pain intensity displayed an L-shaped relationship: SHAP values remained near zero for scores below approximately 5, then increased sharply at higher intensities. For categorical migraine features, the absence of nausea was associated with higher predicted WMH volume than its presence, whereas the presence of somatosensory aura was associated with higher predicted WMH volume relative to its absence (Fig. 3).

### Sensitivity analyses

Multivariable RCS regression confirmed statistically significant non-linearity for attack frequency (P for nonlinear = 0.008) and attack duration (P for nonlinear = 0.02), but not for mean pain intensity (P for nonlinear = 0.54) (Supplementary Table 3; Supplementary Fig. 10). The directions of the categorical associations were also consistent with the main analysis. In the multivariable model, absence of nausea (β = −0.41; 95% CI: -0.71 to -0.12; *P* = 0.006) and presence of somatosensory aura (β = 0.29; 95% CI: 0.04, 0.55; *P* = 0.03) each remained significantly associated with higher WMH volume.

Piecewise linear regression (Supplementary Table 4) showed that for attack frequency and attack duration, plateau-zone slopes above the approximate breakpoints were markedly attenuated relative to the active-zone slopes below (slope reductions: 97.1% and 89.4%). For mean pain intensity, the pattern was reversed: the plateau zone lay below approximately NRS 5 with a near-zero slope, while the active zone above this threshold showed a steep positive slope (slope reduction: 80.1%). All three piecewise models provided better fit than single linear models (ΔAIC = 43.1, 38.1, and 77.6, respectively).

The fully nested feature-selection analysis yielded more conservative estimates (R² = 0.103; AUC = 0.770, 95% CI 0.698–0.842) than the final model, while the same predictors and association patterns were retained. Attack frequency, attack duration, mean pain intensity, and total intracranial volume were selected in all five folds (Supplementary Table 6), and the SHAP dependence patterns were preserved, including the saturating relationships for attack frequency and duration, the threshold pattern for pain intensity, and the directions of the somatosensory-aura and nausea associations (Supplementary Fig. 14). Finally, trial source did not materially affect the results. Adding trial source as a covariate left the key associations essentially unchanged (attack frequency β = 0.031 to 0.031; attack duration β = 0.002 to 0.002; mean pain intensity β = 0.001 to 0.001; nausea β = −0.421 to − 0.417; somatosensory aura β = 0.295 to 0.308), and trial source itself was not associated with WMH volume after adjustment (β = −0.074; *P* = 0.57) (Supplementary Table 7). No interaction between trial source and any of the key migraine predictors was significant (all *P* > 0.05).

### Feature interactions

Pairwise SHAP interaction values were consistently small relative to main effects. The two strongest interactions were age × log-transformed TIV (interaction strength = 0.017, 8.6% of the larger main effect) and attack frequency × attack duration (0.016, 13.9% of the larger main effect) (Supplementary Fig. 11). Colour-stratified dependence plots revealed no discernible patterns of effect modification for either pair (Supplementary Fig. 12**).**

### Patient stratification

**Fig. 4:**
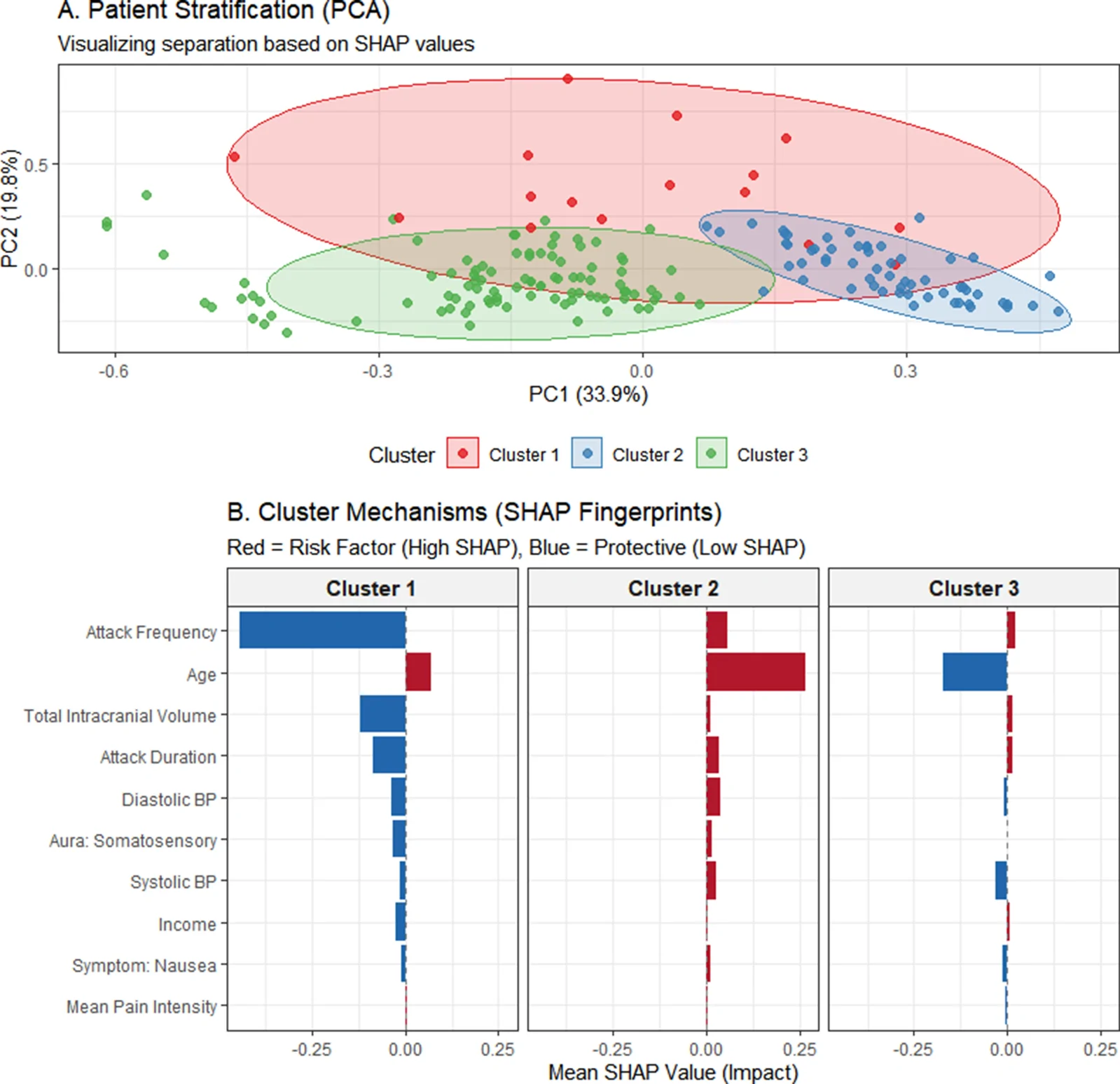
Data-driven migraine phenotypes identified by SHAP-based clustering. (**A**) Principal component analysis (PCA) demonstrating three distinct patient clusters derived from individual SHAP values. (**B**) Mean SHAP fingerprints illustrating the driving features for each cluster. Red bars denote positive contributions to predicted WMH volume (risk factors), and blue bars denote negative contributions (protective factors).Cluster 1 (Age-Dominant Phenotype): Driven primarily by advanced age, with negative contributions from migraine-specific features (e.g., low attack frequency). Cluster 2 (Cumulative Risk Phenotype): Characterized by concurrent positive contributions from both advanced age and high migraine burden, representing a high-risk mixed etiology.Cluster 3 (Migraine-Dominant Phenotype): Comprises younger patients (negative age contribution) where high migraine burden serves as the primary driver of WMH, indicating early-onset structural alterations. Abbreviations: PCA, principal component analysis; SHAP, SHapley Additive exPlanations; WMH, white matter hyperintensity

The elbow method and silhouette coefficient converged on three clusters (Supplementary Fig. 13), well separated in the first two principal components (PC1: 33.9%; PC2: 19.8%) (Fig. 4; cluster characteristics summarised in Supplementary Table 5).

Cluster 1 (Age-Dominant; *n* = 16) was characterised by positive SHAP contributions from age and negative contributions from migraine-related features. Participants had a median age of 57.5 years, infrequent attacks (median 1.0 attack/month), and low WMH volume (mean 0.59 ± 0.62 mL). Cluster 2 (Cumulative Risk; *n* = 59) exhibited concurrent positive contributions from both age and migraine features. This cluster had the highest median age (62.0 years), attack frequency (4.0 attacks/month), attack duration (24.0 h/month), and systolic blood pressure (123.0 mmHg), as well as the highest WMH volume (mean 2.36 ± 2.62 mL). Cluster 3 (Migraine-Dominant; *n* = 96) comprised younger women (median age 43.0 years) with negative SHAP contributions from age but positive contributions from migraine features, and intermediate WMH volume (mean 0.85 ± 0.51 mL). The three phenotypes differed significantly in demographic and clinical characteristics, including age, migraine duration, attack frequency, systolic blood pressure, and somatosensory aura (Supplementary Table 5).

The three-cluster solution showed an average silhouette width of 0.271 (Age-Dominant 0.148; Cumulative-Risk 0.243; Migraine-Dominant 0.308), and bootstrap Jaccard coefficients across 1000 resamples were 0.64, 0.77, and 0.58 for the Age-Dominant, Cumulative-Risk, and Migraine-Dominant clusters, respectively. Within a restricted middle-age band, defined as the interquartile range of age in the sample (38 to 60 years, *n* = 87), all three phenotypes remained represented (*n* = 10, 22, and 55 for the Age-Dominant, Cumulative-Risk, and Migraine-Dominant clusters, respectively), and re-clustering the SHAP profiles of this age-restricted subset reproduced the original assignment closely (adjusted Rand index = 0.92). By contrast, clustering the same participants on the raw values of age, attack frequency, attack duration, and mean pain intensity showed low agreement with the SHAP-based assignment (adjusted Rand index = 0.07), as did a simpler stratification by median age and median severity (adjusted Rand index = 0.29).

### Illustrative individual profiles

**Fig. 5:**
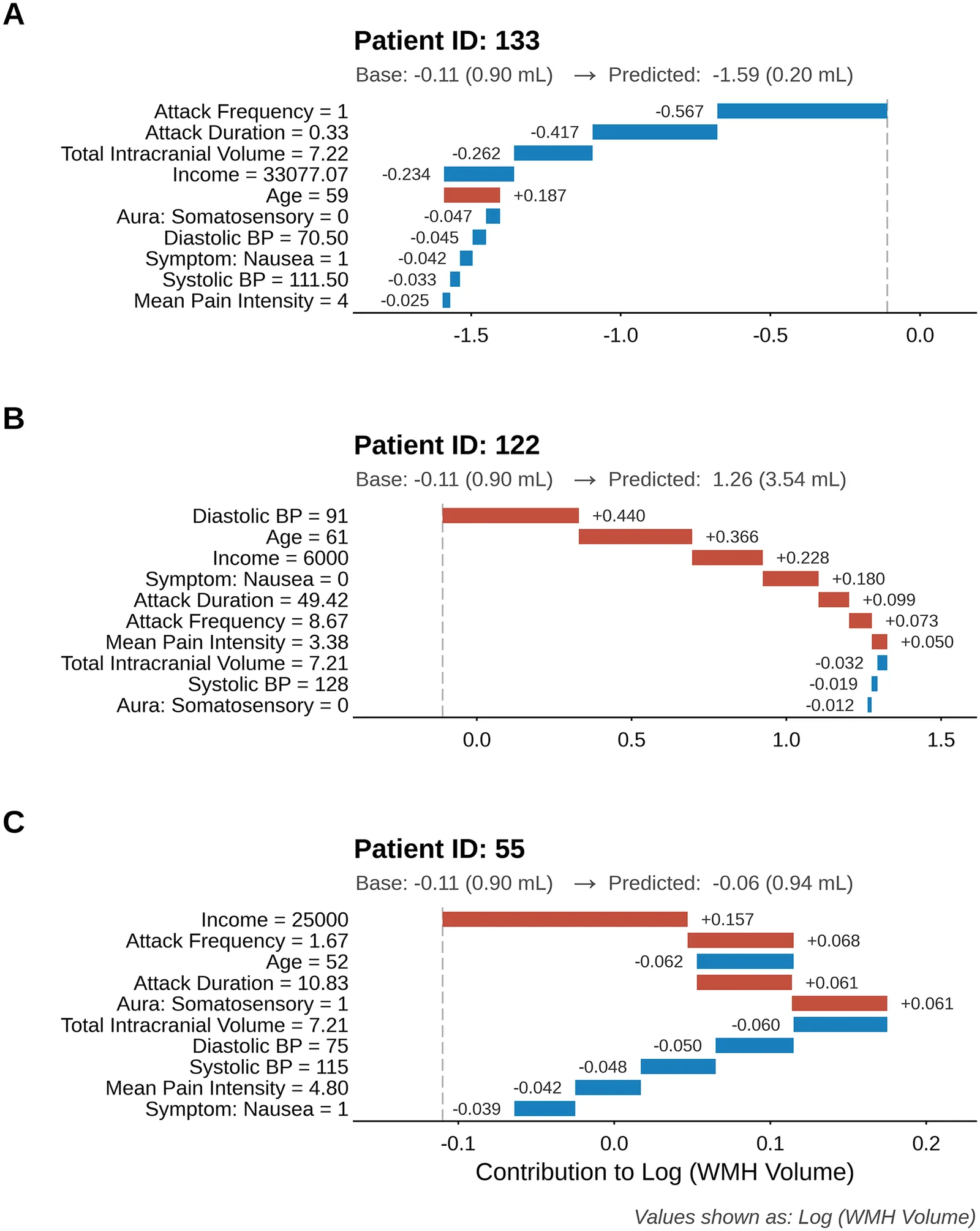
SHAP waterfall plots for representative patients from each cluster. (**A**) Patient 133 (Cluster 1, Age-Dominant). (**B**) Patient 122 (Cluster 2, Cumulative Risk). (**C**) Patient 55 (Cluster 3, Migraine-Dominant).Each horizontal bar represents the SHAP contribution of a single feature to the individual's predicted log-transformed WMH volume. Red bars denote positive contributions (increasing predicted WMH volume); blue bars denote negative contributions (decreasing predicted WMH volume). Features are ordered by the absolute magnitude of their SHAP values. The numerical value adjacent to each bar indicates the SHAP contribution in log(WMH volume) units. Feature values for each patient are displayed on the y-axis labels. The dashed vertical line indicates the base value (−0.11 in log scale), corresponding to the sample mean WMH volume of 0.90 mL. Representative patients were selected based on minimal Euclidean distance to their respective cluster centroids in SHAP feature space.Abbreviations: BP, blood pressure; SHAP, SHapley Additive exPlanations; TIV, total intracranial volume; WMH, white matter hyperintensity

SHAP waterfall plots for one representative participant per cluster illustrate how individual predictions were decomposed into feature-level contributions on the log-transformed scale (Fig. 5**).**

Cluster 1 - Patient 133 (Fig. 5A). This 59-year-old woman had a predicted WMH volume of 0.20 mL (log = − 1.59). Age was the sole positive contributor (SHAP = + 0.187), while low attack frequency (1.0 attack/month; −0.567) and short attack duration (0.33 h/month; −0.417) drove the prediction markedly downward, with additional negative contributions from the remaining features. The profile exemplifies a patient whose age-related risk is substantially offset by a very low migraine burden.

Cluster 2 - Patient 122 (Fig. 5B). This 61-year-old woman had the highest predicted WMH volume in the sample (3.54 mL; log = 1.26). Diastolic blood pressure (91 mmHg; +0.440) was the leading positive contributor, followed by age (+ 0.366). Migraine features added further positive contributions: absence of nausea (+ 0.180), long attack duration (49.42 h/month; +0.099), high attack frequency (8.67 attacks/month; +0.073), and mean pain intensity (3.38; +0.050). The remaining non-migraine features exerted minor effects. This profile exemplifies the Cumulative Risk phenotype, in which advanced age, elevated vascular risk, and high migraine burden converge.

Cluster 3 - Patient 55 (Fig. 5C). This 52-year-old woman had a predicted WMH volume of 0.94 mL (log = − 0.06), near the sample mean. Migraine features were the primary positive contributors: attack frequency (1.67 attacks/month; +0.068), attack duration (10.83 h/month; +0.061), and somatosensory aura (+ 0.061). Age exerted a negative contribution (− 0.062), with additional negative effects from blood pressure, mean pain intensity, and nausea. This profile illustrates a younger patient whose WMH volume is primarily attributable to migraine pathophysiology rather than aging or vascular risk, consistent with the Migraine-Dominant phenotype.

## Discussion

This study applied an explainable machine-learning framework combining XGBoost with SHAP interpretation to 171 women with episodic migraine and yielded three principal findings. First, migraine-specific clinical features were associated with white matter hyperintensity (WMH) burden independently of established vascular risk factors. Second, SHAP dependence analyses revealed non-linear association patterns not previously reported in the migraine-WMH literature, including apparent saturation for attack frequency and duration, a threshold-type pattern for pain intensity, and J-shaped curves for blood pressure. These patterns offer an empirical framework that may help reconcile previously conflicting findings across studies that assumed monotonic, linear relationships. Third, unsupervised SHAP-based clustering identified three data-driven patient phenotypes distinguished by the dominant drivers of their predicted WMH volume, illustrating an approach to risk stratification that moves beyond conventional clinical phenotyping and may inform individualised risk assessment if validated prospectively. These findings should be regarded as exploratory and explanatory rather than causal inference.

Age exhibited a broadly linear positive association with WMH volume and was the single strongest predictor in our model. This is consistent with a large longitudinal study of 15 cohorts that identified age as the dominant predictor of WMH progression [31]. The primacy of age is consistent with the cumulative nature of cerebral small vessel pathology, in which decades of exposure to vascular risk factors, progressive blood-brain barrier dysfunction, and arteriolar remodelling may compound over time and contribute to WMH accumulation [6].

Both systolic and diastolic blood pressure displayed J-shaped relationships with WMH volume, with nadirs in the normotensive range. This bidirectional pattern accords with prior longitudinal evidence that both elevated and low blood pressure are associated with greater WMH burden [32–34]. The ascending limb reflects hypertension-driven arteriolosclerosis and endothelial damage, whereas the descending limb reflects the haemodynamic vulnerability of deep periventricular white matter, an arterial watershed zone supplied by long penetrating end-arterioles, in which chronically low perfusion pressure predisposes to ischaemic change [6, 33, 35, 36]. This vascular susceptibility may provide a plausible context for the migraine-specific associations discussed below.

The inverse association between household income and WMH volume is consistent with epidemiological evidence linking lower socioeconomic status (SES) to greater WMH burden [37], an association likely mediated through the higher prevalence of modifiable vascular risk factors in socioeconomically disadvantaged populations [38].

Turning to migraine-specific features, whether attack frequency and duration are associated with WMH burden has been one of the most contentious questions in the migraine-WMH literature, with studies yielding seemingly irreconcilable results. Evidence of a positive association has emerged from several cohorts. Kruit et al. found that women with high attack frequency had a greater risk of deep white matter lesions in the CAMERA study [3]; Trauninger et al. reported that WMH presence was associated with higher headache frequency [8]; and Ahmed et al. found significantly more frequent attacks in the WMH group [39]. However, other well-designed studies have found no such association. In the ARMR cohort, Chong et al. found no relationship between headache frequency and WMH after accounting for vascular risk factors [40]; and, most notably, the CAMERA-2 nine-year follow-up found that migraine frequency was not associated with lesion progression over time, despite higher baseline deep WMH volume in women with migraine [41].

Our SHAP analysis offers a unifying explanation for these contradictory results. The SHAP contribution of attack frequency rose steeply below approximately 5 attacks/month but then plateaued, and cumulative attack duration showed a similar rapid rise below approximately 24 h/month before levelling off, a saturation pattern corroborated by the sensitivity analyses. Studies assuming linear relationships or using dichotomised outcomes would be unable to detect such saturating associations. Those sampling predominantly from the higher-frequency portion of the curve, where the slope approaches zero, would yield null findings, whereas those capturing the steep initial portion would report positive associations. This may explain why CAMERA-2, following patients with established migraine, found no association of attack frequency with WMH progression, as these patients may already have been on the plateau at baseline.

Mechanistically, and while our data cannot adjudicate between mechanisms, the saturation pattern is compatible with the possibility that the marginal contribution of each additional attack to WMH accumulation diminishes beyond a threshold. Two mechanisms have been proposed in the literature, both of which remain speculative. First, deep white matter has lower blood flow than grey matter and lies within watershed zones supplied by long penetrating end-arterioles that are inherently vulnerable to the prolonged oligaemia accompanying cortical spreading depolarisation (CSD) [42, 43]. Second, CSD-induced ischaemic preconditioning may progressively upregulate endogenous neuroprotective pathways [44–46]. These preconditioning effects would attenuate the white matter consequences of subsequent CSD episodes, producing the observed plateau at higher attack frequencies and longer attack durations.

Mean pain intensity exhibited a distinctive threshold relationship, with SHAP values remaining near zero for Numerical Rating Scale (NRS) scores below approximately 5 and increasing sharply at higher intensities. This non-linear pattern reconciles discrepant findings, with some studies reporting that WMH burden increased with pain intensity [39, 47] and others finding no association [5, 48]. Our analysis indicates that the relationship is not linear, such that mild-to-moderate pain (NRS < 5) may be insufficient to drive measurable WMH accumulation whereas severe pain may be a significant contributor; studies with predominantly mild-to-moderate cohorts or narrow pain ranges would therefore yield null findings. Although multivariable RCS regression did not detect significant non-linearity for pain intensity, this is methodologically expected, as RCS uses smooth cubic polynomials suited to gradual curvature but less sensitive to abrupt step-like transitions, whereas piecewise regression, which can accommodate such transitions, showed the largest improvement in fit among the three migraine features. One possible mechanism, which remains speculative, involves sympathetically mediated haemodynamic stress. Severe pain activates the sympathetic nervous system and the hypothalamic-pituitary-adrenal axis, producing blood pressure surges proportional to pain intensity [49]. Low-to-moderate intensity attacks may generate insufficient sympathetic activation to exceed the autoregulatory capacity of deep white matter arterioles, whereas severe pain may trigger haemodynamic surges that cause transient hyperperfusion and shear-mediated endothelial damage in susceptible microvascular beds [6, 36].

The positive association between somatosensory aura and WMH volume, contrasting with the negligible contribution of visual aura, is a novel finding. Prior research has focused almost exclusively on the dichotomous classification of migraine with versus without aura, with inconsistent results, reporting either no association [11] or a higher frequency of aura in patients with WMH [39]. To our knowledge, no prior study has examined whether specific aura subtypes are differentially associated with WMH burden. Our finding suggests that the topographic extent of CSD, rather than the mere presence of aura, may be more relevant to WMH accumulation. CSD is a slowly propagating wave of near-complete neuronal and glial depolarisation [50]. Because somatosensory aura implies that the CSD wave has propagated beyond the occipital cortex into the parietal somatosensory cortex, it reflects a larger affected cortical territory than isolated visual aura. Experimental evidence supports this mechanism, as Eikermann-Haerter et al. demonstrated that post-CSD oligaemia was significantly deeper at medial compared with lateral cortical areas, inducing ischaemic changes at watershed zones and at the tips of deep penetrating arteries [43]. Given the watershed anatomy of deep white matter, the wider cortical footprint of somatosensory aura would subject a greater volume of vulnerable white matter to prolonged hypoperfusion, whereas isolated visual aura, confined to the occipital cortex, may produce a spatially limited oligaemic zone insufficient to cause measurable change. This may also partly explain prior inconsistent findings, as pooling all aura types would have diluted the signal from somatosensory aura with the null contribution of visual aura.

Counterintuitively, the absence of nausea was associated with higher predicted WMH volume. This contrasts with prior univariable analyses reporting that WMH burden increased with nausea or vomiting [39, 47]. However, our SHAP analysis captures the conditional contribution of nausea after adjusting for pain intensity, attack frequency, blood pressure, and other correlated features, and this direction was reproduced in the leakage-free sensitivity analysis, indicating that it is a stable feature of the fitted model rather than an artefact of feature selection. This nonetheless remains a predictive association rather than a causal effect. One speculative interpretation is that nausea may reflect vagal activation that partially counterbalances the concurrent sympathetic surge, given that the nausea reflex and the baroreceptor reflex share a common brainstem hub in the nucleus tractus solitarius [51], consistent with evidence that vagus nerve stimulation suppresses CSD susceptibility [52] and ameliorates migraine [53]. This mechanism is speculative and is offered only as a hypothesis; residual confounding and the correlation of nausea with other adjusted features cannot be excluded, and the finding should not be read as evidence that nausea protects against WMH. Prospective studies with autonomic function testing would be required to examine it further.

### Clinical and research implications

The saturation patterns for attack frequency and duration place the steepest gradient of predicted WMH volume at relatively modest disease burden, below approximately 5 attacks/month and 24 h/month of cumulative attack duration, with little further change above these values. If confirmed longitudinally, these patterns would suggest that reductions in attack burden may need to extend into or below these ranges before they are associated with meaningfully lower WMH burden, rather than implying a uniform benefit from any partial reduction. The pattern for pain intensity ran in the opposite direction, with the steeper gradient above approximately NRS 5. This pattern raises the hypothesis that preventing escalation to severe pain may be relevant to WMH burden, but this possibility would require prospective evaluation of acute treatment exposure and subsequent WMH change. The differential association of somatosensory but not visual aura, if confirmed, might help to identify patients with greater white matter burden than their overall attack burden would predict, although this is too preliminary to guide management. More immediately, it indicates that recording aura phenomenology in more detail than the current dichotomous classification may be informative for research.

The three SHAP-based phenotypes carry a different kind of implication. Conventional clustering groups patients who resemble one another clinically, whereas clustering on patient-level SHAP values groups patients according to which features drive their predicted WMH volume. The same variables are used, but patients are grouped by the contribution pattern of those variables rather than by their raw values, so two women with similar attack frequency and blood pressure may fall into different phenotypes if their predicted burden is generated in different ways. Each phenotype was therefore characterised by a distinct pattern of feature contributions to predicted WMH volume. In the Age-Dominant phenotype, age contributed positively while migraine features contributed negatively, and these women had infrequent attacks and the lowest WMH volume; intensifying migraine-specific prevention would be unlikely to alter their predicted WMH burden, which was driven mainly by a non-modifiable contributor. The Cumulative-Risk phenotype had concurrent positive contributions from age and migraine features, together with the highest attack frequency, cumulative duration, systolic blood pressure, and WMH volume, suggesting that vascular risk factor management and migraine prevention might both be candidate targets. The Migraine-Dominant phenotype comprised the youngest women, in whom age contributed negatively but migraine features contributed positively; notably, their WMH volume exceeded that of the older Age-Dominant phenotype despite a median age approximately 14 years lower, which is consistent with white matter changes occurring relatively early and suggests that sustained migraine-specific prevention might matter most in this group. These phenotypes are hypotheses rather than validated subgroups. They were derived by unsupervised clustering, one cluster was small (*n* = 16), and although they were not reducible to age and migraine severity alone, whether stratifying by them adds to simple age- and severity-based stratification is untested; future work would need to validate them in independent cohorts and examine whether phenotype-guided intervention improves long-term white matter outcomes.

### Strengths and limitations

Strengths of this study include prospective three-month diary-based ascertainment of migraine features, WMH volumetric quantification on 3T MRI derived from manual or expert-corrected segmentations rather than uncorrected automated output, simultaneous evaluation of more than 30 migraine-specific features, and nested cross-validation with hyperparameter tuning to guard against overfitting. The combination of XGBoost with SHAP enabled detection of non-linear associations and individual-level interpretation not achievable with conventional regression, and key findings were supported by complementary sensitivity analyses, including multivariable restricted cubic spline and piecewise regression models and a fully nested re-estimation of the modelling workflow.

This study has several limitations. First, MRI data were acquired at a single time point, and migraine attack data were collected for only three months, which is an inherent limitation for causal inference. With the model explaining only a modest proportion of the variance (R² = 0.221), much of the variability in WMH volume remains unaccounted for by the included predictors. As such, the present analysis should be interpreted as an exploratory explanatory model rather than a clinical prediction tool, underscoring the need for future studies with expanded predictor sets. Second, the sample was all-female, Hong Kong Chinese, which limits generalizability. Third, the recorded aura prevalence was probably elevated by the ascertainment method. Aura was captured from both a structured interview covering the preceding month and the three-month prospective diary, and specific symptoms were probed within each subtype rather than recording subtype status alone (Supplementary Table 8). Because aura features vary between attacks in the same patient [54], this approach characterises each participant’s aura more completely, but the resulting prevalence is not directly comparable with population surveys based on a single assessment. This, together with recruitment from migraine intervention trials, limit the generalizability of our findings, underscoring the need for larger cohorts and extended longitudinal follow-up of migraine features in future investigations. Fourth, feature selection combined Boruta screening with SHAP-based ranking, so the retained features and their apparent importance could partly reflect this particular sample. Repeating the workflow within each cross-validation fold yielded the same leading predictors and preserved the association patterns, although bias inherent to the selection procedure itself cannot be excluded. Fifth, no formal correction for multiple comparisons was applied. Given the number of features screened and the dependence, interaction, piecewise, and between-cluster comparisons examined, weaker associations in particular should be regarded as hypothesis-generating rather than confirmatory. Sixth, all episodic migraine subtypes were modelled together. Because subtypes may differ in their relationship to WMH, pooling them can obscure subtype-specific patterns and may bias the SHAP-based interpretation, particularly when feature-level contributions are linked to biological mechanisms rather than treated as predictive associations within the fitted model. This interpretive caution is consistent with recent calls for standardized study design and rigorous evaluation of machine-learning research in migraine [55, 56]. Seventh, the piecewise breakpoints were derived from the same SHAP dependence plots and are therefore descriptive rather than independently validated. Finally, the clinical significance of the WMH burden in this exploratory study is unproven, because WMH in migraine may partially differ in natural history and prognostic meaning from vascular WMH [41]. External validation in independent, longitudinal, and multi-ethnic cohorts is needed.

## Conclusions

Using an explainable machine-learning framework, this study identified threshold associations between specific migraine features and WMH volume in women with episodic migraine. The apparent saturation for attack frequency and duration, the L-shaped pattern for pain intensity, and the differential association of somatosensory but not visual aura provide novel insights that may help reconcile previously conflicting findings and that conventional linear analyses would not capture. SHAP-based patient stratification revealed three data-driven phenotypes that may generate hypotheses for future risk-stratification research in migraine. Because the study is cross-sectional and single-centre, these findings are hypothesis-generating and warrant validation in larger, longitudinal, and multi-ethnic cohorts.

## Supplementary Information

Below is the link to the electronic supplementary material.

Supplementary Material 1

## Data Availability

The datasets analysed during the current study are not publicly available but are available from the corresponding author on reasonable request.

## References

[1] Vetvik KG, MacGregor EA (2017) Sex differences in the epidemiology, clinical features, and pathophysiology of migraine. Lancet Neurol 16(1):76–87. 10.1016/S1474-4422(16)30293-9 27836433

[2] Stovner LJ, Nichols E, Steiner TJ, Abd-Allah F, Abdelalim A, Al-Raddadi RM, Ansha MG, Barac A, Bensenor IM, Doan LP, Edessa D, Endres M, Foreman KJ, Gankpe FG, Gopalkrishna G, Goulart AC, Gupta R, Hankey GJ, Hay SI, Hegazy MI, Hilawe EH, Kasaeian A, Kassa DH, Khalil I, Khang Y-H, Khubchandani J, Kim YJ, Kokubo Y, Mohammed MA, Mokdad AH, Moradi-Lakeh M, Nguyen HLT, Nirayo YL, Qorbani M, Ranta A, Roba KT, Safiri S, Santos IS, Satpathy M, Sawhney M, Shiferaw MS, Shiue I, Smith M, Szoeke CEI, Truong NT, Venketasubramanian N, Weldegwergs KG, Westerman R, Wijeratne T, Tran BX, Yonemoto N, Feigin VL, Vos T, Murray CJL (2018) Global, regional, and national burden of migraine and tension-type headache, 1990–2016: a systematic analysis for the Global Burden of Disease Study 2016. The Lancet Neurology 17(11):954–976. 10.1016/S1474-4422(18)30322-3 PMC6191530 30353868

[3] Kruit MC, van Buchem MA, Hofman PA, Bakkers JT, Terwindt GM, Ferrari MD, Launer LJ (2004) Migraine as a risk factor for subclinical brain lesions. JAMA 291(4):427–434. 10.1001/jama.291.4.427 14747499

[4] Bashir A, Lipton RB, Ashina S, Ashina M (2013) Migraine and structural changes in the brain: a systematic review and meta-analysis. Neurology 81(14):1260–1268. 10.1212/WNL.0b013e3182a6cb32 PMC3795609 23986301

[5] Zhang W, Cheng Z, Fu F, Zhan Z (2023) Prevalence and clinical characteristics of white matter hyperintensities in Migraine: A meta-analysis. Neuroimage Clin 37:103312. 10.1016/j.nicl.2023.103312 PMC9827384 36610309

[6] Pantoni L (2010) Cerebral small vessel disease: from pathogenesis and clinical characteristics to therapeutic challenges. Lancet Neurol 9(7):689–701. 10.1016/S1474-4422(10)70104-6 20610345

[7] Debette S, Markus HS (2010) The clinical importance of white matter hyperintensities on brain magnetic resonance imaging: systematic review and meta-analysis. BMJ 341:c3666. 10.1136/bmj.c3666 PMC2910261 20660506

[8] Trauninger A, Leel-Ossy E, Kamson DO, Poto L, Aradi M, Kover F, Imre M, Komaromy H, Erdelyi-Botor S, Patzko A, Pfund Z (2011) Risk factors of migraine-related brain white matter hyperintensities: an investigation of 186 patients. J Headache Pain 12(1):97–103. 10.1007/s10194-011-0299-3 PMC3056006 21331756

[9] Dobrynina LA, Suslina AD, Gubanova MV, Belopasova AV, Sergeeva AN, Evers S, Gnedovskaya EV, Krotenkova MV (2021) White matter hyperintensity in different migraine subtypes. Sci Rep 11(1):10881. 10.1038/s41598-021-90341-0 PMC8149843 34035361

[10] Meilán A, Larrosa D, Ramón C, Cernuda-Morollón E, Martínez-Camblor P, Saiz A, Santamarta E, Pérez-Pereda S, Pascual J (2020) No association between migraine frequency, white matter lesions and silent brain infarctions: a study in a series of women with chronic migraine. Eur J Neurol 27(8):1689–1696. 10.1111/ene.14284 32343863

[11] Gaist D, Garde E, Blaabjerg M, Nielsen HH, Kroigard T, Ostergaard K, Moller HS, Hjelmborg J, Madsen CG, Iversen P, Kyvik KO, Siebner HR, Ashina M (2016) Migraine with aura and risk of silent brain infarcts and white matter hyperintensities: an MRI study. Brain 139(Pt 7):2015–2023. 10.1093/brain/aww099 PMC4939694 27190013

[12] Ashina M (2020) Migraine. N Engl J Med 383(19):1866–1876. 10.1056/NEJMra1915327 33211930

[13] Rajkomar A, Dean J, Kohane I (2019) Machine Learning in Medicine. N Engl J Med 380(14):1347–1358. 10.1056/NEJMra1814259 30943338

[14] Chen T, Guestrin C (2016) XGBoost: a scalable tree boosting system. Proceedings of the 22nd ACM SIGKDD international conference on knowledge discovery and data mining, San francisco, California, USA

[15] Lundberg SM, Lee S-I (2017) A unified approach to interpreting model predictions. Proceedings of the 31st international conference on neural information processing systems, Long Beach, California, USA

[16] Xie YJ, Liao X, Hui SS, Tian L, Yeung WF, Lau AY, Tyrovolas S, Gao Y, Chen X (2023) Tai Chi for the prophylaxis of episodic migraine: protocol of a non-inferiority randomized controlled trial with mechanism exploration. BMC Complement Med Ther 23(1):328. 10.1186/s12906-023-04154-x PMC10507971 37723467

[17] Headache Classification Committee of the International Headache Society (IHS) (2018) The International Classification of Headache Disorders, 3rd edition. Cephalalgia 38(1):1–211. 10.1177/0333102417738202 29368949

[18] von Elm E, Altman DG, Egger M, Pocock SJ, Gøtzsche PC, Vandenbroucke JP (2007) Strengthening the Reporting of Observational Studies in Epidemiology (STROBE) statement: guidelines for reporting observational studies. BMJ 335(7624):806–808. 10.1136/bmj.39335.541782.AD PMC2034723 17947786

[19] Isensee F, Jaeger PF, Kohl SAA, Petersen J, Maier-Hein KH (2021) nnU-Net: a self-configuring method for deep learning-based biomedical image segmentation. Nat Methods 18(2):203–211. 10.1038/s41592-020-01008-z 33288961

[20] Stewart WF, Lipton RB, Kolodner KB, Sawyer J, Lee C, Liberman JN (2000) Validity of the Migraine Disability Assessment (MIDAS) score in comparison to a diary-based measure in a population sample of migraine sufferers. Pain 88(1):41–52. 10.1016/s0304-3959(00)00305-5 11098098

[21] Kosinski M, Bayliss MS, Bjorner JB, Ware JE Jr., Garber WH, Batenhorst A, Cady R, Dahlöf CG, Dowson A, Tepper S (2003) A six-item short-form survey for measuring headache impact: the HIT-6. Qual Life Res 12(8):963–974. 10.1023/a:1026119331193 14651415

[22] Martin BC, Pathak DS, Sharfman MI, Adelman JU, Taylor F, Kwong WJ, Jhingran P (2000) Validity and reliability of the migraine-specific quality of life questionnaire (MSQ Version 2.1). Headache 40(3):204–215. 10.1046/j.1526-4610.2000.00030.x 10759923

[23] Buysse DJ, Reynolds CF 3rd, Monk TH, Berman SR, Kupfer DJ (1989) The Pittsburgh Sleep Quality Index: a new instrument for psychiatric practice and research. Psychiatry Res 28(2):193–213. 10.1016/0165-1781(89)90047-4 2748771

[24] Cohen S, Kamarck T, Mermelstein R (1983) A global measure of perceived stress. J Health Soc Behav 24(4):385–396 6668417

[25] Stekhoven DJ, Bühlmann P (2012) MissForest—non-parametric missing value imputation for mixed-type data. Bioinformatics 28(1):112–118. 10.1093/bioinformatics/btr597 22039212

[26] Kursa MB, Rudnicki WR (2010) Feature Selection with the Boruta Package. J Stat Softw 36(11):1–13. 10.18637/jss.v036.i11

[27] Lundberg SM, Erion G, Chen H, DeGrave A, Prutkin JM, Nair B, Katz R, Himmelfarb J, Bansal N, Lee S-I (2020) From local explanations to global understanding with explainable AI for trees. Nat Mach Intell 2(1):56–67. 10.1038/s42256-019-0138-9 PMC7326367 32607472

[28] Orsini N, Moore A, Wolk A (2022) Interaction Analysis Based on Shapley Values and Extreme Gradient Boosting: A Realistic Simulation and Application to a Large Epidemiological Prospective Study. Front Nutr 9:871768. 10.3389/fnut.2022.871768 PMC9340268 35923201

[29] Frank E, Harrell J (2015) Regression modeling strategies: with applications to linear models, logistic and ordinal regression, and survival analysis, 2nd edn. Springer

[30] Ikotun AM, Ezugwu AE, Abualigah L, Abuhaija B, Heming J (2023) K-means clustering algorithms: A comprehensive review, variants analysis, and advances in the era of big data. Inf Sci 622:178–210. 10.1016/j.ins.2022.11.139

[31] de Kort FAS, Vinke EJ, van der Lelij EJ, Anblagan D, Bastin ME, Beiser A, Brodaty H, Chaturvedi N, Cheng B, Cox SR, DeCarli C, Enzinger C, Fletcher E, Frayne R, de Groot M, Huang F, Ikram MA, Jiang J, Lam BYK, … Biesbroek JM (2025) Cerebral white matter hyperintensity volumes: Normative age- and sex-specific values from 15 population-based cohorts comprising 14,876 individuals. Neurobiology of Aging 146:38–47. 10.1016/j.neurobiolaging.2024.11.006 PMC12087372 39602940

[32] van Dijk EJ, Breteler MMB, Schmidt R, Berger K, Nilsson L-G, Oudkerk M, Pajak A, Sans S, de Ridder M, Dufouil C, Fuhrer R, Giampaoli S, Launer LJ, Hofman A (2004) The Association Between Blood Pressure, Hypertension, and Cerebral White Matter Lesions. Hypertension 44(5):625–630. 10.1161/01.HYP.0000145857.98904.20 15466662

[33] de Leeuw FE, de Groot JC, Oudkerk M, Witteman JC, Hofman A, van Gijn J, Breteler MM (1999) A follow-up study of blood pressure and cerebral white matter lesions. Ann Neurol 46(6):827–833. 10.1002/1531-8249(199912)46:6<827::aid-ana4>3.3.co;2-8 10589534

[34] Kim JS, Lee S, Suh SW, Bae JB, Han JH, Byun S, Han JW, Kim JH, Kim KW (2020) Association of Low Blood Pressure with White Matter Hyperintensities in Elderly Individuals with Controlled Hypertension. J Stroke 22(1):99–107. 10.5853/jos.2019.01844 PMC7005351 32027795

[35] van Dijk EJ, Breteler MM, Schmidt R, Berger K, Nilsson LG, Oudkerk M, Pajak A, Sans S, de Ridder M, Dufouil C, Fuhrer R, Giampaoli S, Launer LJ, Hofman A (2004) The association between blood pressure, hypertension, and cerebral white matter lesions: cardiovascular determinants of dementia study. Hypertension 44(5):625–630. 10.1161/01.Hyp.0000145857.98904.20 15466662

[36] Faraco G, Iadecola C (2013) Hypertension: a harbinger of stroke and dementia. Hypertension 62(5):810–817. 10.1161/HYPERTENSIONAHA.113.01063 PMC3847558 23980072

[37] Morris EP, Turney IC, Palms JD, Zaheed AB, Sol K, Amarante E, Beato J, Chesebro AG, Morales CD, Manly JJ, Brickman AM, Zahodne LB (2023) Racial and ethnic differences in the relationship between financial worry and white matter hyperintensities in Latinx, non-Latinx Black, and non-Latinx White older adults. Neurobiol Aging 129:149–156. 10.1016/j.neurobiolaging.2023.05.008 PMC10878173 37331245

[38] Rodriguez FS, Lampe L, Gaebler M, Beyer F, Baber R, Burkhardt R, Schroeter ML, Engel C, Loffler M, Thiery J, Villringer A, Riedel-Heller SG, Witte AV (2024) Differences in white matter hyperintensities in socioeconomically deprived groups: results of the population-based LIFE Adult Study. Int Psychogeriatr 36(9):785–798. 10.1017/S104161022300025X 37039457

[39] Ahmed SR, Mohamed AAM, Salem HH, Helmy S, Moustafa RR, Borham SMF (2023) Association of white matter hyperintensities with migraine phenotypes and response to treatment. Acta Neurol Belg 123(5):1725–1733. 10.1007/s13760-022-02015-x PMC10505107 35854172

[40] Chong CD, Schwedt TJ, Trivedi M, Chong BW (2022) The Characteristics of White Matter Hyperintensities in Patients With Migraine. Front Pain Res (Lausanne) 3:852916. 10.3389/fpain.2022.852916 PMC9251128 35794956

[41] Palm-Meinders IH, Koppen H, Terwindt GM, Launer LJ, Konishi J, Moonen JM, Bakkers JT, Hofman PA, van Lew B, Middelkoop HA, van Buchem MA, Ferrari MD, Kruit MC (2012) Structural brain changes in migraine. JAMA 308(18):1889–1897. 10.1001/jama.2012.14276 PMC3633206 23150008

[42] Wang Y, Liu G, Hong D, Chen F, Ji X, Cao G (2016) White matter injury in ischemic stroke. Prog Neurobiol 141:45–60. 10.1016/j.pneurobio.2016.04.005 PMC5677601 27090751

[43] Donmez-Demir B, Yemisci M, Uruk G, Soylemezoglu F, Bolbos R, Kazmi S, Dalkara T (2023) Cortical spreading depolarization-induced constriction of penetrating arteries can cause watershed ischemia: A potential mechanism for white matter lesions. J Cereb Blood Flow Metab 43(11):1951–1966. 10.1177/0271678X231186959 PMC10676143 37435741

[44] Viggiano E, Monda V, Messina A, Moscatelli F, Valenzano A, Tafuri D, Cibelli G, De Luca B, Messina G, Monda M (2016) Cortical spreading depression produces a neuroprotective effect activating mitochondrial uncoupling protein-5. Neuropsychiatr Dis Treat 12:1705–1710. 10.2147/NDT.S107074 PMC4946829 27468234

[45] Shen P, Hou S, Zhu M, Zhao M, Ouyang Y, Feng J (2017) Cortical spreading depression preconditioning mediates neuroprotection against ischemic stroke by inducing AMP-activated protein kinase-dependent autophagy in a rat cerebral ischemic/reperfusion injury model. J Neurochem 140(5):799–813. 10.1111/jnc.13922 27987215

[46] Horiguchi T, Snipes JA, Kis B, Shimizu K, Busija DW (2006) Cyclooxygenase-2 mediates the development of cortical spreading depression-induced tolerance to transient focal cerebral ischemia in rats. Neuroscience 140(2):723–730. 10.1016/j.neuroscience.2006.02.025 16567054

[47] Negm M, Housseini AM, Abdelfatah M, Asran A (2018) Relation between migraine pattern and white matter hyperintensities in brain magnetic resonance imaging. Egypt J Neurol Psychiatr Neurosurg 54(1):24. 10.1186/s41983-018-0027-x PMC6133060 30237692

[48] Al-Hashel JY, Alroughani R, Gad K, Al-Sarraf L, Ahmed SF (2022) Risk factors of white matter hyperintensities in migraine patients. BMC Neurol 22(1):159. 10.1186/s12883-022-02680-8 PMC9052543 35488255

[49] Bruehl S, Chung OY (2004) Interactions between the cardiovascular and pain regulatory systems: an updated review of mechanisms and possible alterations in chronic pain. Neurosci Biobehav Rev 28(4):395–414. 10.1016/j.neubiorev.2004.06.004 15341037

[50] Harriott AM, Takizawa T, Chung DY, Chen SP (2019) Spreading depression as a preclinical model of migraine. J Headache Pain 20(1):45. 10.1186/s10194-019-1001-4 PMC6734429 31046659

[51] Benarroch EE (1993) The central autonomic network: functional organization, dysfunction, and perspective. Mayo Clin Proc 68(10):988–1001. 10.1016/s0025-6196(12)62272-1 8412366

[52] Chen SP, Ay I, Lopes de Morais A, Qin T, Zheng Y, Sadeghian H, Oka F, Simon B, Eikermann-Haerter K, Ayata C (2016) Vagus nerve stimulation inhibits cortical spreading depression. Pain 157(4):797–805. 10.1097/j.pain.0000000000000437 PMC4943574 26645547

[53] Silberstein SD, Mechtler LL, Kudrow DB, Calhoun AH, McClure C, Saper JR, Liebler EJ, Rubenstein Engel E, Tepper SJ (2016) Non-Invasive Vagus Nerve Stimulation for the ACute Treatment of Cluster Headache: Findings From the Randomized, Double-Blind, Sham-Controlled ACT1 Study. Headache 56(8):1317–1332. 10.1111/head.12896 PMC5113831 27593728

[54] Hansen JM, Goadsby PJ, Charles AC (2016) Variability of clinical features in attacks of migraine with aura. Cephalalgia 36(3):216–224. 10.1177/0333102415584601 25944814

[55] Petrusic I, Savic A, Mitrovic K, Bacanin N, Sebastianelli G, Secci D, Coppola G (2024) Machine learning classification meets migraine: recommendations for study evaluation. J Headache Pain 25(1):215. 10.1186/s10194-024-01924-x PMC11622592 39639193

[56] Petrusic I, Messina R, Pellesi L, Azorin DG, Chiang CC, Pietra AD, Ha WS, Labastida-Ramirez A, Onan D, Ornello R, Raffaelli B, Rubio-Beltran E, Ruscheweyh R, Tana C, Vuralli D, Waliszewska-Prosol M, Wang W, Wells-Gatnik WD, Martelletti P, Raggi A (2025) Application of machine learning in migraine classification: a call for study design standardization and global collaboration. J Headache Pain 26(1):200. 10.1186/s10194-025-02134-9 PMC12492770 41039195

