# Supplementary material for: Explainable machine learning reveals threshold associations and distinct phenotypes linking migraine features to white matter hyperintensities in women with episodic migraine
Source: J Headache Pain. 2026 Aug 4;27(1):255. doi: 10.1186/s10194-026-02488-8 (PMC13721521; doi:10.1186/s10194-026-02488-8)
Supplement: Supplementary file 1 [file 10194_2026_2488_MOESM1_ESM.docx]

**SUPPLEMENTARY MATERIALS**

[Supplementary Figure 1. Selection of the assistive segmentation model on the manually segmented reference set	2](#_Toc12006)

[Supplementary Figure 2. Distribution of white matter hyperintensity (WMH) volume before and after natural logarithm transformation	3](#_Toc18154)

[Supplementary Figure 3. Feature selection of clinical and demographic variables using the Boruta algorithm	4](#_Toc15492)

[Supplementary Figure 4. Pairwise spearman correlation matrix of selected and migraine features	5](#_Toc6326)

[Supplementary Figure 5. Variance inflation factor analysis for multicollinearity assessment	6](#_Toc11085)

[Supplementary Figure 6. Global feature importance in the initial full XGBoost model	7](#_Toc9080)

[Supplementary Figure 7. Concordance between predicted and observed white matter hyperintensity volumes	8](#_Toc31446)

[Supplementary Figure 8. Receiver operating characteristic curve for high white matter hyperintensity burden classification	9](#_Toc1549)

[Supplementary Figure 9. SHAP dependence plots illustrating the associations between clinical and demographic features and predicted white matter hyperintensity volume	10](#_Toc32163)

[Supplementary Figure 10. Adjusted non-linear associations of attack frequency and attack duration with log-transformed WMH volume estimated by restricted cubic splines	11](#_Toc9118)

[Supplementary Figure 11. Global feature interaction strengths in the final model	12](#_Toc16932)

[Supplementary Figure 12. SHAP interaction dependence plots for the top ranked feature pairs	13](#_Toc20768)

[Supplementary Figure 13. Determination of the optimal number of clusters for k-means clustering	14](#_Toc20660)

[Supplementary Figure 14. Leakage-free SHAP dependence plots from the fully nested feature-selection sensitivity analysis	15](#_Toc18383)

[Supplementary Table 1. Summary of missing data across study variables	16](#_Toc20136)

[Supplementary Table 2. Summary of variables excluded from the analysis due to near-zero variance	17](#_Toc19602)

[Supplementary Table 3. Univariable and multivariable linear regression with restricted cubic spline analysis for the 10 selected predictors of log-transformed WMH volume	18](#_Toc22182)

[Supplementary Table 4. Piecewise linear regression quantifying plateau effects for attack frequency, attack duration, and mean pain intensity	19](#_Toc15207)

[Supplementary Table 5. Characteristics of participants stratified by clustered migraine phenotype	20](#_Toc6362)

[Supplementary Table 6. Feature-selection stability in the fully nested sensitivity analysis	21](#_Toc24395)

[Supplementary Table 7. Key WMH associations from the multivariable linear regression model, without and with trial source as a covariate	22](#_Toc10702)

[Supplementary Table 8. Prevalence of aura subtypes by ascertainment source	23](#_Toc564)

# Supplementary Figure 1. Selection of the assistive segmentation model on the manually segmented reference set

To select the model used to assist manual delineation, four segmentation methods (LST-LGA, LST-LPA, BIANCA, and nnU-Net) were trained and compared on the 30 manually segmented reference scans under nested five-fold cross-validation (inner loop for hyperparameter tuning; outer loop for performance estimation). (A) Dice similarity coefficient (DSC). (B) Sensitivity. (C) Precision. Each dot represents one scan; box plots show the median and interquartile range, and the open circle denotes the mean. P values are from pairwise comparisons of nnU-Net with each alternative method. nnU-Net showed the highest agreement with the manual reference standard and was therefore chosen to generate the initial predictions that were subsequently manually corrected for the remaining scans. This figure informs model selection only; all WMH masks used in the analyses were manual or expert-corrected.

# Supplementary Figure 2. Distribution of white matter hyperintensity (WMH) volume before and after natural logarithm transformation

Left: Raw WMH volume (mL) showing a right-skewed distribution with a long positive tail. The solid orange line indicates the mean and the dotted yellow line indicates the median; the separation between the two reflects the degree of skewness. Right: Log-transformed WMH volume (log[WMH volume]) showing an approximately symmetric distribution. The pink dashed curve represents the theoretical normal distribution fitted to the transformed data. After transformation, the mean and median converge, and the empirical density curve closely approximates the theoretical normal curve, supporting the use of log(WMH volume) as the outcome variable in subsequent analyses.

Abbreviation: WMH, white matter hyperintensity.

# Supplementary Figure 3. Feature selection of clinical and demographic variables using the Boruta algorithm

Boxplots represent the importance (Z-score) of clinical and demographic variables for predicting log-transformed white matter hyperintensity (WMH) volume. Green boxplots indicate "confirmed" features that performed significantly better than random shadow attributes. Red boxplots indicate "rejected" non-significant features, and yellow boxplots denote "tentative" features. The vertical dashed line represents the maximum Z-score of the shadow attributes (Shadow Max), serving as the threshold for selection. Only confirmed and tentative variables were retained for subsequent predictive modeling.

# Supplementary Figure 4. Pairwise spearman correlation matrix of selected and migraine features

The heatmap illustrates Spearman correlation coefficients (r) among the features selected by the Boruta algorithm, alongside total intracranial volume and migraine-specific variables. Red shading denotes positive correlations, whereas blue shading denotes negative correlations, with color intensity proportional to the correlation strength. Dendrograms on the top and left axes indicate hierarchical clustering of features based on their correlation profiles. Variables exhibiting high collinearity (|r| ≥0.8 ) were scrutinized prior to the modeling process to prevent multicollinearity.

# Supplementary Figure 5. Variance inflation factor analysis for multicollinearity assessment

The dot plot illustrates Variance Inflation Factor (VIF) values for all candidate predictors. The vertical dashed red line indicates the conservative threshold of VIF = 5. All selected variables exhibited values well below this threshold, confirming the absence of severe multicollinearity.

# Supplementary Figure 6. Global feature importance in the initial full XGBoost model

SHapley Additive exPlanations (SHAP) summary plot ranking all candidate predictors according to their mean absolute SHAP values. Each dot represents a single patient. The color gradient indicates the raw feature value, ranging from low (yellow) to high (purple). The x-axis displays the SHAP value, which quantifies the impact of each feature on the model's output; positive values indicate an increased predicted WMH volume. Vertical dispersion (jitter) at a single SHAP value indicates a high density of overlapping data points.

# Supplementary Figure 7. Concordance between predicted and observed white matter hyperintensity volumes

Violin plots illustrate the distribution of predicted vs. observed log-transformed WMH volumes across quartiles (Q1–Q4) of the observed data. Internal white boxplots represent the median and interquartile range (IQR). Black dots and the connecting line represent mean predicted values. The model shows a significant increasing trend (P for trend <0.001) and a Pearson correlation of

r=0.473, indicating good concordance between predicted and observed WMH burdens.

# Supplementary Figure 8. Receiver operating characteristic curve for high white matter hyperintensity burden classification

The curve evaluates the final model's discriminative ability to classify patients with a "high WMH burden," defined a priori as the top quartile (top 25%) of observed WMH volumes. The solid orange line represents the mean ROC curve derived from cross-validation, and the shaded area indicates the standard deviation across the cross-validation folds.

Abbreviations: AUC, area under the curve; CI, confidence interval.

# Supplementary Figure 9. SHAP dependence plots illustrating the associations between clinical and demographic features and predicted white matter hyperintensity volume

SHAP dependence plots demonstrating the independent predictive contributions of clinical and demographic features to WMH volume. The y-axis represents the SHAP value (the feature's adjusted contribution to the model output), where values >0 indicate an increased predicted WMH volume. Solid red lines represent smoothing curves fitted by generalized additive models (GAMs) with 95% confidence intervals. Marginal density distributions (blue shaded areas) and rug marks are displayed at the bottom.

Abbreviations: SHAP, SHapley Additive exPlanations; WMH, white matter hyperintensity; BP, blood pressure; TIV, log-transformed total intracranial volume.

# Supplementary Figure 10. Adjusted non-linear associations of attack frequency and attack duration with log-transformed WMH volume estimated by restricted cubic splines

Solid red curves represent the adjusted change in log-transformed WMH volume (Δ log [WMH Volume]) relative to the reference value (observed minimum for each predictor), estimated from a multivariable ordinary least squares model with restricted cubic splines (4 knots) for all continuous predictors, adjusting for age, household income, diastolic and systolic blood pressure, total intracranial volume, mean pain intensity, nausea, and somatosensory aura. Shaded bands denote 95% confidence intervals. Dashed horizontal lines indicate zero (no change from reference). Histograms at the bottom of each panel show the sample distribution of the predictor variable; n = frequency count. The x-axis for attack duration (right panel) is displayed on a square-root scale to improve visualisation of the right-skewed distribution. Attack frequency was truncated at the 95th percentile of the sample distribution.

# Supplementary Figure 11. Global feature interaction strengths in the final model

The bar chart displays the mean absolute SHAP interaction values for the top identified feature pairs, sorted by strength. The "Ratio" (in parentheses) quantifies the relative magnitude of the interaction, calculated as the interaction strength divided by the maximum main effect strength of the two features.

# Supplementary Figure 12. SHAP interaction dependence plots for the top ranked feature pairs

**The vertical dispersion of points at any given x-value represents the interaction effect. The lack of distinct vertical stratification or color-based separation (e.g., high values consistently appearing above low values) visually confirms that interaction effects are weak. The risk trajectories are primarily dictated by the main effects of individual features rather than their interactions with secondary variables.**

# Supplementary Figure 13. Determination of the optimal number of clusters for k-means clustering

(A) The Elbow method evaluates the total within-cluster sum of squares (WSS) across varying numbers of clusters (k). (B) The Silhouette method calculates the average silhouette width for each k. The vertical dashed lines in both panels indicate the selected optimal number of clusters (k=3), corresponding to the "elbow" point in (A) and the maximum silhouette width in (B).

# Supplementary Figure 14. Leakage-free SHAP dependence plots from the fully nested feature-selection sensitivity analysis

SHAP values were obtained from the nested cross-validation in which the entire feature-selection pipeline was repeated within the training partition of each outer fold; each observation is displayed using the out-of-fold model that did not use it for feature selection or training. (A) Attack frequency (attacks/month), (B) attack duration (hours/month), and (C) mean pain intensity (Numerical Rating Scale) are shown as SHAP dependence plots with a fitted trend, shaded 95% confidence band, and a marginal density of the predictor; (D) nausea and (E) somatosensory aura are shown as SHAP distributions by symptom status.

# Supplementary Table 1. Summary of missing data across study variables

| **Variable** | **Missing Values** | |
| --- | --- | --- |
|  | **Number** | **Percentage** |
| Monthly family income | 49 | 0.287 |
| Pittsburgh Sleep Quality Index (PSQI) score | 5 | 0.029 |
| Percent Body Fat | 2 | 0.012 |
| Diastolic Blood Pressure | 1 | 0.006 |
| Systolic Blood Pressure | 1 | 0.006 |
| Waist Circumference | 1 | 0.006 |
| Hip Circumference | 1 | 0.006 |
| Thigh Circumference | 1 | 0.006 |
| Age at Menarche | 1 | 0.006 |
| Daily Fruit Intake (servings) | 1 | 0.006 |
| Daily Vegetable Intake (servings) | 1 | 0.006 |
| Daily Whole Grain Intake (servings) | 1 | 0.006 |

Variables not listed have no missing values.

# Supplementary Table 2. Summary of variables excluded from the analysis due to near-zero variance

| **Variable** | **Majority Class** | **Minority Class** | **Frequency Ratio** |
| --- | --- | --- | --- |
| **Medical History** |  |  |  |
| Cardiovascular Disease | 164 | 7 | 23.43 |
| Angina pectoris | 169 | 2 | 84.5 |
| Stroke | 166 | 5 | 33.2 |
| Liver Disease | 170 | 1 | 170 |
| Breast Cancer | 163 | 8 | 20.38 |
| Uterus Cancer | 170 | 1 | 170 |
| Chronic lung disease | 171 | 0 | - |
| Ovarian cancer | 171 | 0 | - |
| Family History of Breast Cancer | 163 | 8 | 20.38 |
| Family History of Uterus Cancer | 170 | 1 | 170 |
| Family History of Colon Cancer | 168 | 3 | 56 |
| **Reproductive History** |  |  |  |
| History of infertility | 163 | 8 | 20.38 |
| Hormonal medication use | 167 | 4 | 41.75 |
| History of abortion | 169 | 2 | 84.5 |
| **Lifestyle Factors** |  |  |  |
| Smoking Status | 167 | 4 | 41.75 |
| **Migraine Features** |  |  |  |
| Hyperactivity | 167 | 4 | 41.75 |
| Sleep Trigger | 163 | 8 | 20.38 |
| Environmental Trigger | 163 | 8 | 20.38 |

The frequency ratio is defined as the ratio of the frequency of the most common value to the frequency of the second most common value. Variables with a frequency ratio greater than the threshold of 19 (corresponding to a 95/5 split) were excluded to ensure model stability.

# Supplementary Table 3. Univariable and multivariable linear regression with restricted cubic spline analysis for the 10 selected predictors of log-transformed WMH volume

| **Variable** | **Uni β (95% CI)** | **Uni P** | **Multi β (95% CI)** | **Multi P (linear)** | **P overall (RCS)** | **P nonlinear** |
| --- | --- | --- | --- | --- | --- | --- |
| Age (years) | 0.024 (0.014, 0.033) | < 0.001 | 0.019 (0.010, 0.029) | < 0.001 | 0.002 | 0.62 |
| Monthly household income, (1000 HKD) | -0.011 (-0.017, -0.004) | 0.001 | -0.009 (-0.014, -0.003) | 0.005 | 0.03 | 0.50 |
| Attack Frequency (attacks/month) | 0.058 (0.015, 0.102) | 0.009 | 0.032 (-0.010, 0.074) | 0.14 | 0.008 | 0.008 |
| Diastolic blood pressure (mmHg) | 0.025 (0.012, 0.039) | < 0.001 | 0.015 (-0.002, 0.033) | 0.09 | 0.24 | 0.52 |
| Total intracranial volume (mL) | 0.574 (-1.273, 2.421) | 0.54 | 2.127 (0.454, 3.801) | 0.01 | 0.046 | 0.39 |
| Attack Duration (hours/month) | 0.000 (-0.003, 0.004) | 0.77 | -0.001 (-0.004, 0.002) | 0.43 | 0.04 | 0.02 |
| Systolic blood pressure (mmHg) | 0.015 (0.007, 0.024) | < 0.001 | 0.002 (-0.009, 0.013) | 0.76 | 0.15 | 0.07 |
| Mean Pain Intensity | 0.041 (-0.041, 0.124) | 0.33 | 0.005 (-0.069, 0.079) | 0.90 | 0.74 | 0.54 |
| Nausea | -0.302 (-0.625, 0.021) | 0.07 | -0.412 (-0.705, -0.118) | 0.006 | 0.006 | — |
| Somatosensory Aura | 0.393 (0.123, 0.664) | 0.005 | 0.292 (0.038, 0.547) | 0.03 | 0.03 | — |

Uni β, unstandardised coefficient from univariable linear regression;

Multi β, unstandardised coefficient from multivariable linear regression including all 10 predictors simultaneously;

P overall (RCS), likelihood-ratio test for the overall association of the predictor with log-transformed WMH volume in a multivariable model with restricted cubic splines (4 knots at Harrell's recommended percentiles) for all continuous predictors, with the reference value set to the observed minimum;

P nonlinear, likelihood-ratio test comparing the full restricted cubic spline model to a nested model retaining only the linear term for that predictor, testing whether the non-linear (spline) components significantly improve model fit;

CI, confidence intervall; WMH, white matter hyperintensity.

# Supplementary Table 4. Piecewise linear regression quantifying plateau effects for attack frequency, attack duration, and mean pain intensity

| **Metric** | **Frequency (≥ 5/month)** | **Duration (≥ 24 h)** | **Intensity (≤ 5)** |
| --- | --- | --- | --- |
| Active-zone slope (SE) | 0.1092 (0.0116) | 0.0128 (0.0018) | 0.0378 (0.0046) |
| Plateau-zone slope (SE) | 0.0032 (0.0012) | −0.0014 (0.0006) | −0.0075 (0.0025) |
| Plateau slope p | 0.011 | 0.017 | 0.003 |
| Slope reduction | 97.10% | 89.40% | 80.10% |
| SHAP range in plateau / total SHAP range | 4.00% | 9.60% | 14.90% |
| Plateau R² | 0.135 | 0.105 | 0.076 |
| ΔAIC (piecewise − linear) | 43.1 | 38.1 | 77.6 |

Breakpoints were pre-specified from SHAP dependence analysis (attack frequency: 5 attacks/month; attack duration: 24 hours/month; mean pain intensity: NRS 5).

Slope reduction = 1 − |plateau slope / active-zone slope|.

ΔAIC = AIC_piecewise − AIC_linear; positive values indicate that the piecewise model provides superior fit over a single linear model.

AIC, Akaike information criterion; NRS, numerical rating scale; SE, standard error; SHAP, SHapley Additive exPlanations.

# Supplementary Table 5. Characteristics of participants stratified by clustered migraine phenotype

|  |  | Phenotype |  | **P value** |
| --- | --- | --- | --- | --- |
|  | Age-Dominant | Cumulative Risk | Migraine-Dominant |  |
| No. of Participants | 16 | 59 | 96 |  |
| Age (years) | 57.50 (50.75, 60.50) | 62.00 (58.50, 64.00) | 43.00 (30.00, 51.00) | <0.001 |
| Monthly household income, HKD | 30836.93 (20000.00, 34573.08) | 30000.00 (20000.00, 36176.13) | 30000.00 (20000.00, 37007.74) | 0.99 |
| Frequency (attacks/month) | 1.00 (0.46, 1.00) | 4.00 (2.33, 7.83) | 3.00 (1.92, 4.67) | <0.001 |
| Diastolic blood pressure (mmHg) | 72.00 (69.00, 76.00) | 72.50 (67.25, 80.75) | 72.50 (65.00, 79.00) | 0.612 |
| Total intracranial volume (mL) | 7.22 (7.17, 7.23) | 7.22 (7.17, 7.28) | 7.25 (7.20, 7.29) | 0.036 |
| Duration (hours/month) | 8.92 (3.00, 14.62) | 24.00 (10.14, 49.65) | 19.35 (9.75, 37.04) | 0.002 |
| Nausea, n (%) | 13 (81.2) | 41 (69.5) | 78 (81.2) | 0.219 |
| Systolic blood pressure (mmHg) | 117.25 (109.62, 131.25) | 123.00 (111.25, 133.00) | 112.00 (104.00, 122.12) | <0.001 |
| Somatosensory aura, n (%) | 2 (12.5) | 33 (55.9) | 38 (39.6) | 0.005 |
| Intensity (0-10) | 5.00 (3.75, 6.00) | 4.28 (3.26, 5.66) | 4.31 (3.50, 5.63) | 0.646 |
| WMH volume (mL) | 0.59 (0.62) | 2.36 (2.62) | 0.85 (0.51) | <0.001 |

Data are presented as median (interquartile range) for continuous variables and n (%) for categorical variables.

P values were calculated using the Kruskal–Wallis test for continuous variables and the Chi-square test/Fisher's exact test for categorical variables.

# Supplementary Table 6. Feature-selection stability in the fully nested sensitivity analysis

| **Feature** | **Folds selected (of 5)** |
| --- | --- |
| Attack frequency (attacks/month) | 5 |
| Attack duration (hours/month) | 5 |
| Mean pain intensity | 5 |
| Total intracranial volume | 5 |
| Diastolic blood pressure | 4 |
| Age | 3 |
| Hormonal trigger | 3 |
| Nausea | 3 |
| Somatosensory aura | 3 |
| Household income | 2 |
| Menopausal status | 2 |
| Photophobia | 2 |
| Systolic blood pressure | 2 |
| Stress trigger | 2 |
| Physical-activity trigger | 1 |
| Hypertension history | 1 |
| Speech difficulty | 1 |
| Perceived stress score | 1 |

The table lists the number of outer cross-validation folds (of five) in which each feature was retained after the complete selection pipeline was repeated independently within the training partition of each fold.

# Supplementary Table 7. Key WMH associations from the multivariable linear regression model, without and with trial source as a covariate

| **Predictor** | **β (without trial)** | **P value** | **β (with trial)** | **P value** |
| --- | --- | --- | --- | --- |
| Attack frequency (attacks/month) | 0.031 | 0.154 | 0.031 | 0.15 |
| Attack duration (hours) | 0.002 | 0.645 | 0.002 | 0.66 |
| Mean pain intensity (0-10) | 0.001 | 0.969 | 0.001 | 0.97 |
| Nausea (present vs absent) | −0.421 | 0.005 | −0.417 | 0.006 |
| Somatosensory aura (present vs absent) | 0.295 | 0.023 | 0.308 | 0.02 |
| Trial source (RCT 1 vs RCT 2) | – | – | −0.074 | 0.57 |

Values are unstandardised regression coefficients (β) for log-transformed WMH volume from the multivariable linear regression model including all selected predictors (Attack frequency, Attack duration, Mean pain intensity, Somatosensory aura, Nausea, age, systolic blood pressure, diastolic blood pressure, total intracranial volume, monthly family income ) as independent variables, before and after adding trial source as a covariate.

# Supplementary Table 8. Prevalence of aura subtypes by ascertainment source

| Aura subtype | Prospective diary (3 months) | Structured interview (preceding 12 months) | Either source |
| --- | --- | --- | --- |
| Visual | 62 (36.3) | 62 (36.3) | 84 (49.1) |
| Somatosensory | 52 (30.4) | 43 (25.1) | 73 (42.7) |
| Motor | 26 (15.2) | 8 (4.7) | 29 (17.0) |
| Cognitive | 30 (17.5) | 28 (16.4) | 48 (28.1) |

Data are n (%); N = 171. A subtype was coded as present if endorsed by either source, and the combined variables were used in all analyses.
